# G protein–coupled estrogen receptor 1 ameliorates nonalcoholic steatohepatitis through targeting AMPK-dependent signaling

**DOI:** 10.1016/j.jbc.2024.105661

**Published:** 2024-01-20

**Authors:** Longlong Li, Yao Yao, Yulei Wang, Ji Cao, Zhihao Jiang, Ying Yang, Huihui Wang, Haitian Ma

**Affiliations:** 1 Key Laboratory of Animal Physiology and Biochemistry, College of Veterinary Medicine, Nanjing Agricultural University, Nanjing, China; 2 MOE Joint International Research Laboratory of Animal Health and Food Safety, College of Veterinary Medicine, Nanjing Agricultural University, Nanjing, China; 3 State Key Laboratory of Natural Medicines, Key Laboratory of Drug Metabolism and Pharmacokinetics, China Pharmaceutical University, Nanjing, China

**Keywords:** G protein-coupled estrogen receptor 1, AMP-activated protein kinase, hepatocyte, insulin resistance, nonalcoholic steatohepatitis

## Abstract

Nonalcoholic fatty liver disease (NAFLD), especially nonalcoholic steatohepatitis (NASH), has emerged as a prevalent cause of liver cirrhosis and hepatocellular carcinoma, posing severe public health challenges worldwide. The incidence of NASH is highly correlated with an increased prevalence of obesity, insulin resistance, diabetes, and other metabolic diseases. Currently, no approved drugs specifically targeted for the therapies of NASH partially due to the unclear pathophysiological mechanisms. G protein–coupled estrogen receptor 1 (GPER1) is a membrane estrogen receptor involved in the development of metabolic diseases such as obesity and diabetes. However, the function of GPER1 in NAFLD/NASH progression remains unknown. Here, we show that GPER1 exerts a beneficial role in insulin resistance, hepatic lipid accumulation, oxidative stress, or inflammation *in vivo* and *in vitro*. In particular, we observed that the lipid accumulation, inflammatory response, fibrosis, or insulin resistance in mouse NAFLD/NASH models were exacerbated by hepatocyte-specific GPER1 knockout but obviously mitigated by hepatic GPER1 activation in female and male mice. Mechanistically, hepatic GPER1 activates AMP-activated protein kinase signaling by inducing cyclic AMP release, thereby exerting its protective effect. These data suggest that GPER1 may be a promising therapeutic target for NASH.

Nonalcoholic fatty liver disease (NAFLD) is a comprehensive term that includes a range of conditions, from simple fat accumulation in the liver (nonalcoholic fatty liver) to inflammation and fibrosis (nonalcoholic steatohepatitis), which is a prevalent cause of chronic liver diseases (1) and poses severe public health problems that affect the health of approximately 1.7 billion people worldwide (2, 3). Without effective treatment, nonalcoholic fatty liver (NAFL) can further progress to nonalcoholic steatohepatitis (NASH), which is characterized by severe liver damage, including hepatic steatosis with severe fibrosis and inflammation (4). About one-fifth of individuals with NAFLD progress to NASH, which is considered a precursor to advanced liver diseases such as hepatocellular carcinoma (5). Early studies summarized the pathogenesis of NAFLD as the “two-hit hypothesis”, that is, the accumulation of lipids within the hepatocytes' cytoplasm (the first hit) triggers subsequent cytotoxic events (the second hit), which further contribute to NAFLD progression (6). Recent studies found that genetic polymorphism and epigenetics are the key pathogenic factors in NAFLD progression, and the “multiple hit hypothesis” has gradually been widely recognized (7). Although these two hypotheses are slightly different, hepatic lipid excessive accumulation and systemic metabolism disorders are crucial to the occurrence and development of NAFLD, which clearly points out the potential therapeutic strategy for anti-NAFLD. Although a series of in-depth studies have been carried out on the etiology and relevant pathophysiological mechanisms of NAFLD, there are currently no U.S. Food and Drug Administration and the European Medicines Agency–approved pharmaceutical treatments specifically for NAFLD. Hence, it is urgent to probe the potential target and develop therapeutic drugs to halt or reverse of NAFLD progression.

It is worth noting that there are notable gender differences in both the severity and prevalence of NAFLD. In general, the prevalence and severity of NAFLD in premenopausal women between the ages of 20 to 50 is largely lower than that of men with the same age and also significantly lower than postmenopausal women (8, 9). These observations prompted researchers to speculate that the decrease in circulating estrogen level was the leading cause for the obvious difference in the prevalence of NAFLD between premenopausal and postmenopausal women. Accumulating evidence has demonstrated that estrogen deficiency can develop insulin resistance and fatty liver in animal models (10), which was alleviated by treatment with estrogens, predominantly 17β-estradiol (E2) (11). The beneficial effects of E2 on a variety of metabolic diseases, including NAFLD and insulin resistance, are largely attributed to its binding to classical nuclear estrogen receptors (ER) α and β (12), although rapid signaling mediated by E2 has also been acknowledged as essential for systemic estrogenic activities (13). Numerous studies have demonstrated that nuclear ERs’ (mainly ERα) signaling exerts a crucial role in the benefits of estrogen on hepatic steatosis challenged by a high-fat diet (HFD), and ERα is deemed as an important target for the anti-NAFLD (14, 15). However, it remains unknown whether the membrane ER exerts a crucial action in NAFLD progression.

Unlike the classical nuclear ER, G protein-coupled estrogen receptor 1 (GPER1; also known as GPER or GPR30) has been identified as a new type of ER of Gαs protein–coupled receptor family, which belongs to the nonclassical membrane ER and acts a variety of roles in regulating metabolism and immunity by inducing the rapid generation of second messengers such as cycle AMP and Ca^2+^ in response to E2 (16, 17). However, some studies question the role of GPER1 as a direct target of estrogen (18, 19). GPER1 can coordinate or independently mediate multiple signal pathways with classical nuclear ER, thereby regulating the transcription and translation of multiple genes, which leads to controversial research based on ERs. Tamoxifen (TAM), a selective ER modulator, as anti-estrogenic agent that is clinically applicable to breast cancer and long-term TAM administration results in steatohepatitis (20). However, a recent study indicated that short-term TAM treatment protects against fatty liver in mice (21). This paradoxical phenomenon may be attributed to differences in treatment time, administration method, and animal gender. Importantly, TAM is also an agonist of GPER1, which may be involved in the regulatory effect of TAM on fatty liver. Recently, we reported that activation of GPER1 mediates the estrogenic effect of dehydroepiandrosterone in the prevention of lipid metabolism disorders and inflammation in female mice (22). However, further research is needed on the role of GPER1 in NASH progression.

In this study, we used various methods to construct metabolic disorder models in female and male mice to comprehensively elucidate the role and potential mechanism of GPER1 in regulating fatty liver. We found that GPER1 acts a crucial role in the metabolic disorders, including obesity, insulin resistance, and hepatic lipid accumulation in female mice and plays a crucial effect in the protective effect of E2 against palmitic acid (PA) and oleic acid (OA) mixture (PO)-challenged lipid accumulation, oxidative stress, and inflammation in hepatocytes. Moreover, hepatocyte-specific GPER1 knockout (GPER1-HKO) in female and male mice exacerbated HFD- or high-fat and high-cholesterol (HFHC) diet-induced lipid accumulation, inflammatory response, fibrosis, or insulin resistance in mouse NAFLD/NASH models. In contrast, GPER1 activation prevents the progression of HFD- or HFHC diet-induced NAFLD/NASH models in female and male mice. Mechanistically, hepatic GPER1 signaling activates AMP-activated protein kinase (AMPK) by inducing cyclic AMP release, thereby exerting its protective effect. These data suggest that GPER1 could be developed as a potential target for anti-NASH.

## Results

### GPER1 protects against estrogen deficiency–induced obesity, insulin resistance, hepatic lipid accumulation, and inflammatory response in female mice

To determine the potential involvement of GPER1 in estrogen deficiency–induced obesity, insulin resistance, and hepatic lipid accumulation, the OVX female mice in which bilateral surgical removal of the ovaries were treated with GPER1-specific agonist G1 at dose of 5 mg/kg/2 days by gavage for 20 weeks. G1 displays an affinity for GPER1 of approximately 10 nM with no significant binding or function at either ERα or ERβ at concentrations as high as 1 to 10 μM (23, 24). As expected, no significant differences in systemic metabolic profiles, including body weight, insulin resistance, lipid accumulation, and inflammation were observed between normal chow (NC) diet-fed mice by treatment with vehicle and G1 (Fig. S1). However, the OVX-induced increases in the body weight, white adipose tissue (WAT) weight, and the ratio of WAT weight to body weight were significantly inhibited by treatment with G1 (Fig. S1, *A* and *B*). Accordingly, the fasting blood glucose levels, insulin levels, and homeostatic model assessment-insulin resistance (HOMA-IR) values were significantly increased in Vehicle-OVX female mice compared to the Vehicle-Sham female mice, and the G1-OVX female mice showed a markedly lower fasting blood glucose levels, insulin levels, and HOMA-IR values compared to the Vehicle-OVX female mice (Fig. S1, *C*–*E*). Moreover, G1 treatment markedly alleviated OVX-induced compromises in glucose tolerance and insulin sensitivity in the Vehicle-Sham female mice, as indicated by intraperitoneal glucose tolerance test (GTT) and insulin tolerance test (ITT) assays (Fig. S1, *F* and *G*). Periodic acid-Schiff staining results showed that G1 treatment remarkably attenuated the OVX-induced decrease of glycogen contents in the Vehicle-Sham female mice (Fig. S1*H*). Consistently, OVX led to impaired insulin signaling as evidenced by the markedly decreased expression levels of phosphorylated IRS1^Tyr608^, AKT^Ser473^, and GSK3β in the livers of female mice, which was memorably ameliorated by G1 (Fig. S1*I*). In addition to the tendency for attenuating insulin resistance and obesity, we also found that administration of G1 attenuated OVX-induced increases in liver weight (Fig. S1*J*) and excessive lipid accumulation, as evidenced by the levels of triglycerides (TG) and total cholesterol (TC) in the livers (Fig. S1*K*) and serum (Fig. S1*L*) as well as H&E (Fig. S1*M*) and Oil red O staining (Fig. S1*N*), accompanied by markedly decreased expression levels of genes related to the uptake, transport, and synthesis of fatty acids and significantly increased expression levels of genes participating in fatty acid β-oxidation in G1-OVX female mice compared with Vehicle-OVX female mice (Fig. S1*O*). Furthermore, inflammatory cell infiltration into the liver (Fig. S1*P*) was substantially mitigated by treatment with G1 compared with Vehicle-OVX mice, accompanied by decreased pro-inflammatory factors expression levels (Fig. S1*Q*). Together, these data demonstrate that activation of GPER1 alleviates estrogen deficiency–caused obesity, insulin resistance, hepatic lipid accumulation, and inflammation in female mice.

**Figure 1:**
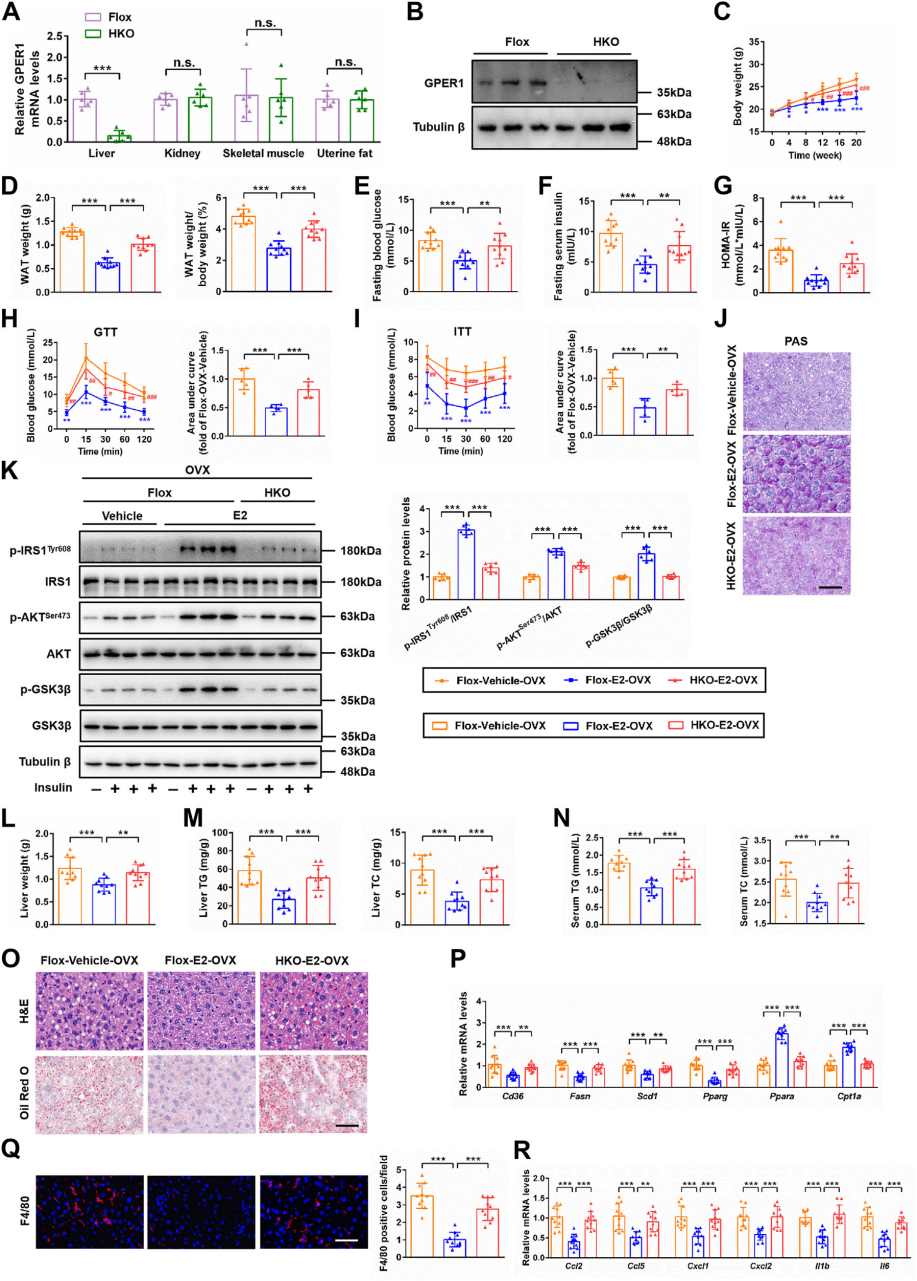
**Hepatocyte-specific GPER1 knockout reverses the protective effects of estradiol against insulin resistance, hepatic lipid accumulation, and inflammation in OVX female mice.***A*, relative GPER1 mRNA levels in the livers, kidneys, skeletal muscles, and uterine fats from GPER1-Flox control and GPER1-HKO female mice (n = 6 mice/group). *B*, representative Western blot image of GPER1 protein level in the livers from GPER1-Flox control and GPER1-HKO female mice (n = 3 mice/group). Tubulin β was served as the loading control. *C*, body weight of the GPER1-HKO and GPER1-Flox control female mice underwent bilateral ovariectomized (OVX) with/without subcutaneous implantation of 17β-estradiol (E2) sustained release tablet, accompanied by normal chow (NC) diet consumption for 0- to 20-week period (n = 10 mice/group). *D*, white adipose tissue (WAT) weight and ratios of WAT weight to body weight of the OVX female mice in the indicated group (n = 10 mice/group). *E*–*G*, fasting blood glucose levels (*E*), fasting blood insulin levels (*F*), and HOMA-IR values (*G*) of the OVX female mice in the indicated group (n = 10 mice/group). *H* and *I*, blood glucose levels after treatment with vehicle or E2 for 20 continuous weeks in NC diet–fed GPER1-HKO OVX female mice and their corresponding controls GPER1-Flox OVX female mice during intraperitoneal GTT (*H*) and intraperitoneal ITT (*I*). The corresponding areas under the curve are indicated on the *right* (n = 5 mice/group). *J*, representative images of periodic acid-Schiff (PAS) staining on the liver sections of the OVX female mice in the indicated group (n = 6 mice/group). Scale bar represents 50 μm. *K*, immunoblotting analyses of total and phosphorylated IRS1 (Tyr608), AKT (Ser473), and GSK3β protein levels in response to an intraperitoneal injection of saline or insulin (1.0 IU/kg for 15 min) in the liver tissues of the OVX female mice in the indicated group (n = 6 mice/group). Tubulin β was served as the loading control. The immunoblot was quantified on the *right*. *L*, liver weight of the OVX female mice in the indicated group (n = 10 mice/group). *M*, hepatic triglyceride (TG) and total cholesterol (TC) contents of the OVX female mice in the indicated group (n = 10 mice/group). *N*, serum TG and TC contents of the OVX female mice in the indicated group (n = 10 mice/group). *O*, representative images of H&E (*upper*) and Oil Red O staining (*lower*) on the liver sections of the OVX female mice in the indicated group (n = 6 mice/group). Scale bar represents 50 μm. *P*, relative mRNA levels of factors related to fatty acid metabolism in the livers of the OVX female mice in the indicated group (n = 10 mice/group). *Q*, representative images of F4/80 (*red*) immunofluorescence staining on the liver sections of the OVX female mice in the indicated group, in which nuclei were stained with DAPI (*blue*). F4/80 positive cells in each field were quantified on the *right* (n = 10 mice/group). *R*, relative mRNA levels of pro-inflammatory factors in the livers of the OVX female mice in the indicated group (n = 10 mice/group). In all statistical plots, data are expressed as the mean ± SD. For statistical analysis, a two-tailed Student’s *t* test was used for (*A*), and one-way ANOVA with Bonferroni analysis was used for (*C*–*I*), (*K*–*N*), (*P*) and (*R*). The mRNA expression of target genes was normalized to that of Actb. For (*C*), (*H*) (*left*), and (*I*) (*left*), ∗*p* < 0.05, ∗∗*p* < 0.01, ∗∗∗*p* < 0.001, Flox-E2-OVX group *versus* Flox-Vehicle-OVX group; ^#^*p* < 0.05, ^##^*p* < 0.01, ^###^*p* < 0.001, HKO-E2-OVX group *versus* Flox-E2-OVX group. For (*A*), (*D*–*G*), (*H*) (*right*), (*I*) (*right*), (*K*–*N*), (*P*) and (*R*), ∗∗*p* < 0.01, ∗∗∗*p* < 0.001, comparison between the indicated groups; n.s., no significance, *p* ≥ 0.05, comparison between the indicated groups. GPER1, G protein–coupled estrogen receptor 1; GTT, glucose tolerance test; HOMA-IR, homeostatic model assessment-insulin resistance; ITT, insulin tolerance test.

The liver is the major metabolic organ and plays an important role in mediating the regulation of estrogen in metabolic homeostasis. Therefore, we subsequently generated the hepatic-specific GPER1 knockout (GPER1-HKO) mouse to certify the role of hepatic GPER1 in the protective effect of estrogen against metabolic disorders in OVX female mice with or without subcutaneous implantation of E2-sustained release tablet. Depletion of GPER1 in the livers of GPER1-HKO female mice was confirmed by Western blot and RT-qPCR analyses (Fig. 1, *A* and *B*). Hepatic GPER1 depletion largely reversed the protective roles of E2 on the OVX-induced body weight gain (Fig. 1*C*), WAT gain (Fig. 1*D*), glucose homeostasis impairment, and insulin resistance (Fig. 1, *E*–*K*), liver weight gain (Fig. 1*L*), hepatic and serum lipid metabolism disorders (Fig. 1, *M*–*P*), and hepatic inflammation (Fig. 1, *Q* and *R*) in female mice, which indicate that hepatic GPER1 involved in the protective effects of E2 on OVX-induced metabolic diseases. Taken together, GPER1 protects against estrogen deficiency–induced obesity, insulin resistance, hepatic lipid accumulation, and inflammatory response in female mice.

### GPER1 is involved in the protective effect of estrogen against lipid accumulation, oxidative stress, and inflammation in hepatocytes under metabolic stress

**Figure 2:**
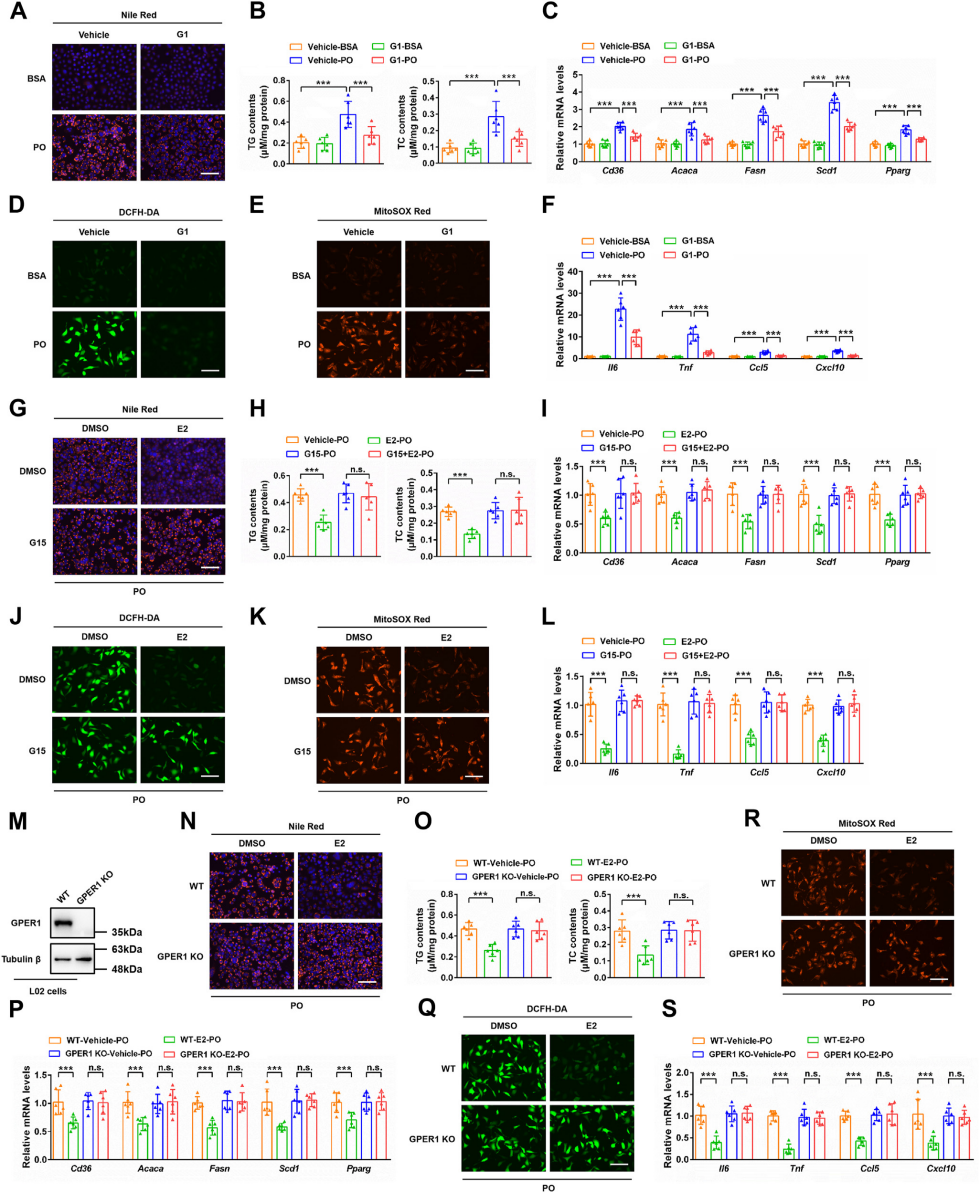
**GPER1 plays a crucial role in the protective effects of estradiol on lipid accumulation, oxidative stress, and inflammation in L02 cells under metabolic stress.***A*, representative images of Nile Red staining in L02 cells treated with vehicle or GPER1-specific agonist G1 (100 nM) followed by BSA or PO (palmitic acid and oil acid mixture) stimulation for 12 h (n = 3 independent experiments). Scale bar represents 100 μm. *B*, triglyceride (TG) and total cholesterol (TC) contents in L02 cells from the indicated group (n = 6). *C*, relative mRNA levels of factors related to fatty acid metabolism in L02 cells from the indicated group (n = 6). *D*, representative images of DCFH-DA probe–stained L02 cells in the indicated group (n = 3 independent experiments). Scale bar represents 100 μm. *E*, representative images of MitoSOX Red probe–stained L02 cells in the indicated group (n = 3 independent experiments). Scale bar represents 100 μm. *F*, relative mRNA levels of factors related to inflammatory response in L02 cells from the indicated group (n = 6). *G*, representative images of Nile Red staining of L02 cells challenged by PO and treated with vehicle, 17β-estradiol (E2; 10 nM), GPER1 antagonist G15 (10 μM), or E2 in combination with G15 (n = 3 independent experiments). Scale bar represents 100 μm. *H*, TG and TC contents in L02 cells from the indicated group (n = 6). *I*, relative mRNA levels of factors related to fatty acid metabolism in L02 cells from the indicated group (n = 6). *J*, representative images of DCFH-DA probe–stained L02 cells in the indicated group (n = 3 independent experiments). Scale bar represents 100 μm. *K*, representative images of MitoSOX Red probe–stained L02 cells in the indicated group (n = 3 independent experiments). Scale bar represents 100 μm. *L*, relative mRNA levels of factors related to inflammatory response in L02 cells from the indicated group (n = 6). *M*, immunoblotting analysis of GPER1 protein level in the WT and GPER1 KO L02 cells (n = 3 independent experiments). *N*, representative images of Nile Red staining in the WT and GPER1 KO L02 cells challenged by PO and cotreated with vehicle or E2 (n = 3 independent experiments). Scale bar represents 100 μm. *O*, TG and TC contents in L02 cells from the indicated group (n = 6). *P*, relative mRNA levels of factors related to fatty acid metabolism in L02 cells from the indicated group (n = 6). *Q*, representative images of DCFH-DA probe–stained L02 cells in the indicated group (n = 3 independent experiments). Scale bar represents 100 μm. *R*, representative images of MitoSOX Red probe–stained L02 cells in the indicated group (n = 3 independent experiments). Scale bar represents 100 μm. *S*, relative mRNA levels of factors related to inflammatory response in L02 cells from the indicated group (n = 6). In all statistical plots, data are expressed as the mean ± SD and analyzed by one-way ANOVA with Bonferroni analysis. ∗∗∗*p* < 0.001, comparison between the indicated groups; n.s., no significance, *p* ≥ 0.05, comparison between the indicated groups. The mRNA expression of target genes was normalized to that of Actb. BSA, bovine serum albumin; GPER1, G protein–coupled estrogen receptor 1; PO, palmitic acid-oleic acid mixture.

To explore the effect of GPER1 on lipid accumulation under metabolic stress, the PO-stimulated L02 cells with GPER1 overexpression (Fig. S2*A*) were generated by transfection with a vector carrying the GPER1 gene. Surprisingly, no significant difference in lipid accumulation was observed between the PO-stimulated L02 cells transfected with the control vector (NC overexpression) and the GPER1 overexpression vector (GPER1 overexpression) (Fig. S2, *B*–*D*). On the contrary, in the presence of estrogen (the ligand of GPER1), overexpression of GPER significantly reduces PO-induced lipid accumulation in hepatocytes (Fig. S2, *B*–*D*). These results were further confirmed in HepG2 cells by the same transfection method (Fig. S2, *E*–*H*). Interestingly, we found that the L02 cells treated with GPER1-specific agonist G1 exhibit a lower lipid accumulation (Fig. 2, *A* and *B*) and oxidative stress (Fig. 2, *D* and *E*) compared to the PO-induced hepatocytes. Moreover, GPER1 activation markedly mitigated PO-induced upregulation of factors related to the transport and synthesis of fatty acids (Fig. 2*C*) and pro-inflammatory mediators (Fig. 2*F*) in L02 cells. Similar results were also found in HepG2 cells that activation of GPER1 alleviates PO-induced lipid accumulation, oxidative stress, and inflammatory response (Fig. S3, *A*–*E*). These results suggest that activation of GPER1, rather than merely enhancing its protein expression level, acts a crucial role in lipid accumulation, oxidative stress, and inflammatory response of hepatocytes.

To further explore whether GPER1 is involved in the regulation of E2 on metabolic disorders, the hepatocytes were pretreated with GPER1 antagonist G15 or GPER1 knockout. Notably, G15 pretreatment completely dispelled E2-mediated inhibition on lipid accumulation, oxidative stress, and inflammatory response in PO-induced L02 cells (Fig. 2, *G*–*L*) and HepG2 cells (Fig. S3, *F*–*J*). Moreover, GPER1 knockout (GPER1 KO) L02 cells (Fig. 2*M*) also exhibited a complete elimination of the inhibitory effects of E2 on lipid accumulation, oxidative stress, and inflammation (Fig. 2, *N*–*S*). Similar results were also found in GPER1 KO HepG2 cells (Fig. S3*K*) and primary hepatocytes (Fig. S2*Q*) isolated from GPER1-HKO female mice that GPER1 participated in the inhibitory effects of E2 on lipid accumulation, oxidative stress, or inflammation (Fig. S3, *L*–*P* and *R*–*T*). Overall, these data demonstrate that GPER1 plays a crucial role in the beneficial effects of E2 on lipid accumulation, oxidative stress, and inflammatory response in hepatocytes under metabolic stress.

Considering that both GPER1 and nuclear ERs mediate the beneficial effects of E2 on metabolic disorders, we further investigated the contribution of nuclear ERs, including ERα and ERβ, to the beneficial metabolic outcomes observed in PO-induced L02 cells and HepG2 cells. Our results showed that the knockdown of ERα (Fig. S4, *A* and *E*) by transfection with siRNA targeting ERα completely blocked the inhibitory effects of E2 on lipid accumulation and oxidative stress in PO-induced L02 cells (Fig. S4, *B*–*D*) and HepG2 cells (Fig. S4, *F*–*H*). On the contrary, the inhibitory effects of E2 on lipid accumulation and oxidative stress in PO-induced L02 cells (Fig. S5, *A*–*C*) and HepG2 cells (Fig. S5, *D*–*F*) were not significantly changed after treatment with PHTPP, a ERβ inhibitor. Therefore, ERα, rather than ERβ, is involved in estrogen-mediated protection against PO-induced hepatic lipid accumulation and oxidative stress in hepatocytes. Overall, these data demonstrate that there may be an interaction between GPER1 and ERα that coordinates the beneficial effects of estrogen on lipid accumulation, oxidative stress, and inflammatory response in hepatocytes under metabolic stress. However, the mutual regulatory relationship between GPER1 and nuclear ERs and its beneficial regulatory effects on metabolic outcomes remain to be further studied.

### Hepatocyte-specific GPER1 deletion aggravates HFD-induced insulin resistance, hepatic steatosis, and inflammation in female mice

**Figure 3:**
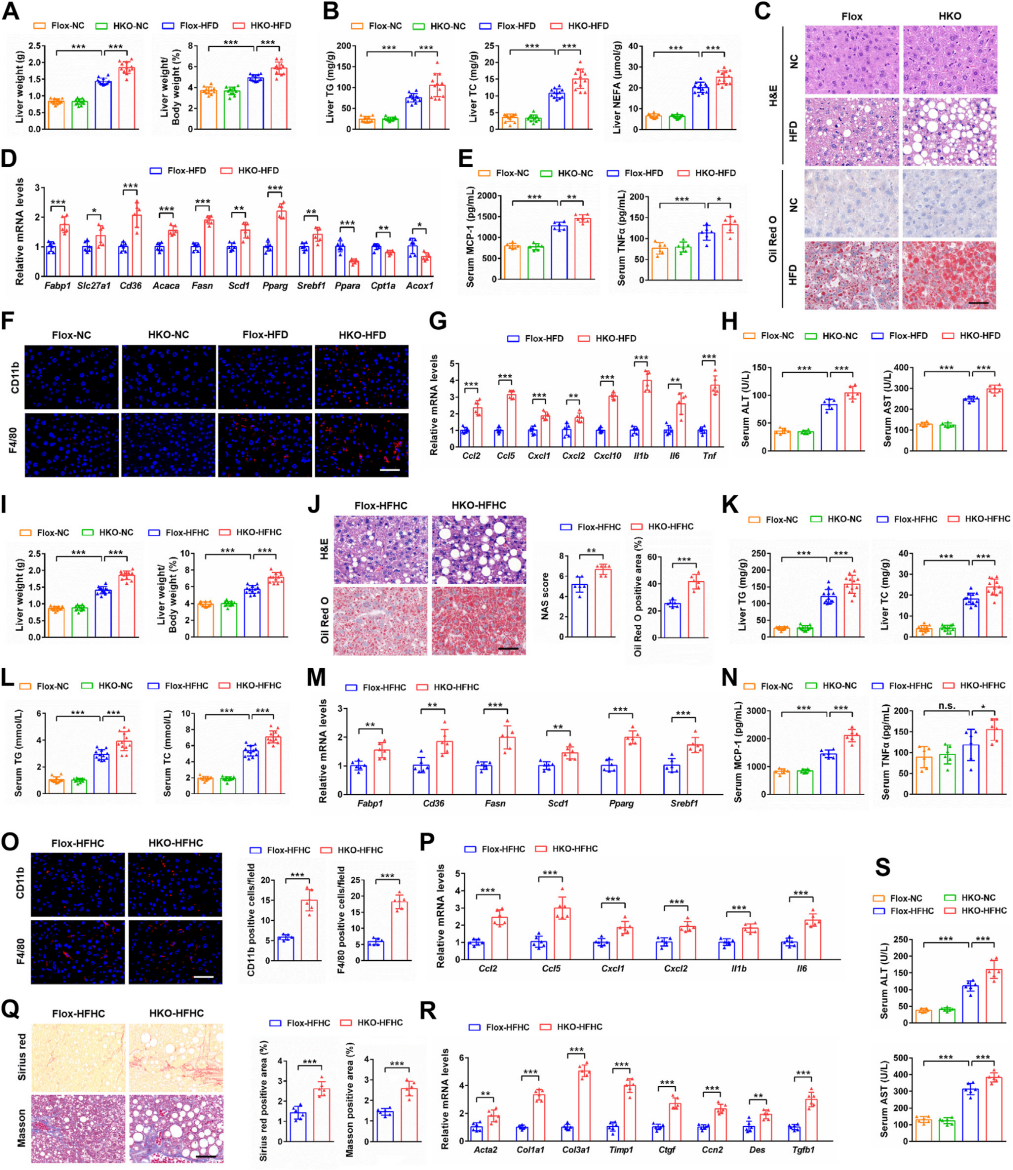
**Hepatocyte-specific GPER1 knockout exacerbates the hepatic steatosis, inflammation, or fibrosis induced by HFD/HFHC diet in female mice.***A*, liver weight and ratios of liver weight to body weight of the GPER1-HKO female mice and their corresponding controls GPER1-Flox female mice after normal chow (NC) diet or high-fat diet (HFD) consumption for 24 weeks (n = 12 mice/group). *B*, hepatic triglyceride (TG), total cholesterol (TC), and nonesterified fatty acid (NEFA) contents of the female mice in the indicated group (n = 12 mice/group). *C*, representative H&E (*upper*) and Oil Red O (*lower*) staining on the liver sections of the female mice in the indicated group (n = 6 mice/group). Scale bar represents 50 μm. *D*, relative mRNA levels of factors related to fatty acid metabolism in the livers of the female mice in the indicated group (n = 6 mice/group). *E*, serum concentration of cytokines MCP-1 and TNFα of the female mice in the indicated group (n = 6 mice/group). *F*, representative images of CD11b (*red*, *upper*) and F4/80 (*red*, *lower*) immunofluorescence staining on the liver sections of the female mice in the indicated group, in which nuclei were stained with DAPI (*blue*) (n = 6 mice/group). Scale bar represents 50 μm. *G*, relative mRNA levels of pro-inflammatory mediators in the livers of the female mice in the indicated group (n = 6 mice/group). *H*, serum alanine aminotransferase (ALT) and aspartate aminotransferase (AST) activities of the female mice in the indicated group (n = 6 mice/group). *I*, liver weight and ratios of liver weight to body weight of the GPER1-HKO female mice and their corresponding controls GPER1-Flox mice after NC or high-fat and high-cholesterol (HFHC) diet consumption for 20 weeks (n = 12 mice/group). *J*, representative H&E (*upper*) and Oil Red O (*lower*) staining on the liver sections of the female mice in the indicated group (n = 6 mice/group). Scale bar represents 50 μm. NAS score and Oil Red O positive area were quantified respectively on the *right*. *K*, hepatic TG and TC contents of the female mice in the indicated group (n = 12 mice/group). *L*, serum TG and TC contents of the female mice in the indicated group (n = 12 mice/group). *M*, relative mRNA levels of factors related to fatty acid metabolism in the livers of the female mice in the indicated group (n = 6 mice/group). *N*, serum concentration of cytokines MCP-1 and TNFα of the female mice in the indicated group (n = 6 mice/group). *O*, representative images of CD11b (*red*, *upper*) and F4/80 (*red*, *lower*) immunofluorescence staining on the liver sections of the female mice in the indicated group, in which nuclei were stained with DAPI (*blue*) (n = 6 mice/group). Scale bar represents 50 μm. CD11b and F4/80 positive cells in each field were quantified respectively on the *right*. *P*, relative mRNA levels of pro-inflammatory mediators in the livers of the female mice in the indicated group (n = 6 mice/group). *Q*, representative images of Sirius red (*upper*) and Masson (*lower*) staining on the liver sections of the female mice in the indicated group (n = 6 mice/group). Scale bar represents 50 μm. Sirius red and Masson positive area were quantified respectively on the *right*. *R*, relative mRNA levels of profibrotic factors in the livers of the female mice in the indicated group (n = 6 mice/group). *S*, serum ALT and AST activities of the female mice in the indicated group (n = 6 mice/group). In all statistical plots, data are expressed as the mean ± SD. For statistical analysis, a two-tailed Student’s *t* test was used for (*D*), (*G*), (*J*), (*M*), and (*O*–*R*), and one-way ANOVA with Bonferroni analysis was used for (*A*), (*B*), (*E*), (*H*), (*I*), (*K*), (*L*), (*N*), and (*S*). The mRNA expression of target genes was normalized to that of Actb. ∗*p* < 0.05, ∗∗*p* < 0.01, ∗∗∗*p* < 0.001, comparison between the indicated groups. GPER1, G protein–coupled estrogen receptor 1; HFD, high fat diet.

Estrogen has multiple beneficial effects in metabolic disease through binding with estrogen receptors in target tissues (25). It is well known that metabolic diseases are often closely related to the occurrence and development of NAFLD (26). Based on the findings that GPER1 acts a crucial role in the protective effects of estrogen on obesity, insulin resistance, hepatic lipid accumulation, and inflammatory response, we speculated that hepatic GPER1 may be involved in the progression of NAFLD. To this end, the hepatocyte-specific GPER1 knockout (GPER1-HKO) female mouse was generated and then fed with an NC diet or HFD for 24 weeks. No significant differences in systemic metabolic profiles, including liver weight, WAT weight, body weight, glucose homeostasis, insulin resistance, lipid accumulation, and inflammation were observed between GPER1-Flox female mice and GPER1-HKO female mice fed with the NC diet (Figs. 3 and S6). However, with HFD consumption for 24 weeks, the GPER1-HKO female mice showed higher body weight, liver weight, and ratios of liver weight to body weight than the GPER1-Flox control female mice (Figs. 3*A* and S6*A*). Accordingly, the GPER1-HKO female mice also exhibited higher WAT weight and ratios of WAT weight to body weight than the GPER1-Flox control female mice (Fig. S6*B*). Moreover, the GPER1-HKO female mice developed more severe glucose homeostasis impairment and insulin resistance, as indicated by the elevation of fasting blood glucose levels, insulin levels, and HOMA-IR values, the reduction of glycogen levels, and more impaired insulin signaling pathway, as well as intraperitoneal GTT and ITT assays than that of the GPER1-Flox control female mice (Fig. S6, *C*–*I*). In addition, lipid accumulation in the livers and serum was markedly increased in the GPER1-HKO female mice compared to the GPER1-Flox control female mice (Figs. 3*B* and S6*J*). In line with lipid accumulation, H&E and Oil red O staining results also indicated that hepatic GPER1 deletion led to more severe hepatic steatosis than that of the GPER1-Flox control female mice, accompanied by memorably enhanced mRNA levels of genes related to the uptake, transport, and synthesis of fatty acids and remarkably reduced expression levels of genes participating in fatty acid β-oxidation (Figs. 3, *C* and *D* and S6*K*). These results indicate that GPER1 acts an important role in HFD-induced insulin resistance and hepatic steatosis. Systemic chronic inflammation acts an important role in the occurrence and development of NAFLD (27). Therefore, the inflammatory response was evaluated and compared in GPER1-Flox control female mice and GPER1-HKO female mice fed with HFD for 24 weeks. The GPER1-HKO female mice displayed higher serum pro-inflammatory mediator levels and severe inflammatory cell infiltration into the liver than the GPER1-Flox control female mice under HFD consumption for 24 weeks (Figs. 3, *E* and *F*, and S6*L*). Consistently, hepatic GPER1 deletion results in the elevation of pro-inflammatory factors mRNA levels in the livers (Fig. 3*G*). Finally, more severe hepatic injury was observed in the GPER1-HKO female mice than that in the GPER1-Flox control female mice, as evidenced by higher serum alanine aminotransferase (ALT) and aspartate aminotransferase (AST) levels (Fig. 3*H*). Taken together, these results suggest that hepatocyte-specific GPER1 deletion exacerbates the progression of NAFLD in female mice.

### GPER1-HKO exacerbates HFHC-induced hepatic steatosis, inflammation, and fibrosis in female and male mice

To further evaluate whether GPER1 also acts a crucial role in the progression of female NASH, the female mice were fed with HFHC diet for 20 weeks to establish a NASH female model, which exhibited more severe inflammatory response and fibrosis than the NAFLD model fed with HFD. Consistent with the profound alterations of phenotype in NAFLD female mouse fed with HFD for 24 weeks, the GPER1-HKO female mice fed with HFHC diet showed higher liver weight and ratios of liver weight to body weight than the GPER1-Flox control female mice (Fig. 3*I*). Moreover, H&E and Oil Red O staining assays indicated that hepatic GPER1 knockout largely exacerbated hepatic steatosis than the GPER1-Flox control female mice after HFHC feeding (Fig. 3*J*). Meantime, lipid accumulation in the livers and serum remarkably increased in GPER1-HKO female mice than that of the GPER1-Flox control female mice in response to HFHC consumption (Fig. 3, *K* and *L*). Consistently, the GPER1-HKO female mice fed with the HFHC diet also exhibited higher mRNA levels of genes participating in the uptake, transport, and synthesis of fatty acids than that of the GPER1-Flox control female mice (Fig. 3*M*). Furthermore, HFHC diet–induced inflammation was markedly aggravated by hepatic GPER1 depletion, as indicated by the analysis of serum pro-inflammation markers concentration and hepatic staining of F4/80 and CD11b, as well as the mRNA levels of pro-inflammatory factors in the livers of female mice (Fig. 3, *N*–*P*). These results imply that hepatic GPER1 acts an important role in HFHC-induced hepatic steatosis and inflammation in female mice. Next, fibrosis indicators in NASH were measured (28). We found that hepatic GPER1 depletion led to more severe fibrosis, as evidenced by the hepatic staining of Sirius red and Masson and the profibrotic factors mRNA expression levels in the livers of female mice (Fig. 3, *Q* and *R*). Similarly, hepatic GPER1 depletion also aggravated HFHC diet–induced liver injury, as showed by the enhanced serum ALT and AST in female mice (Fig. 3*S*). Considering that male mice have lower circulating estrogen levels and are more sensitive to estrogen, GPER1 may also have an important role in NASH male mice. To further evaluate whether GPER1 also acts a crucial role in the progression of male NASH, the male mice were fed with an HFHC diet for 16 weeks to establish a NASH male model, and we found that GPER1-HKO male mice exhibited more severe hepatic steatosis, inflammation, and fibrosis than that of the GPER1-Flox control male mice (Fig. S7). Taken together, these results suggest that hepatocyte-specific GPER1 deletion aggravates NASH progression in female and male mice.

### Targeted activation of hepatic GPER1 mitigates HFD- and HFHC diet-induced NAFLD/NASH in female and male mice

**Figure 4:**
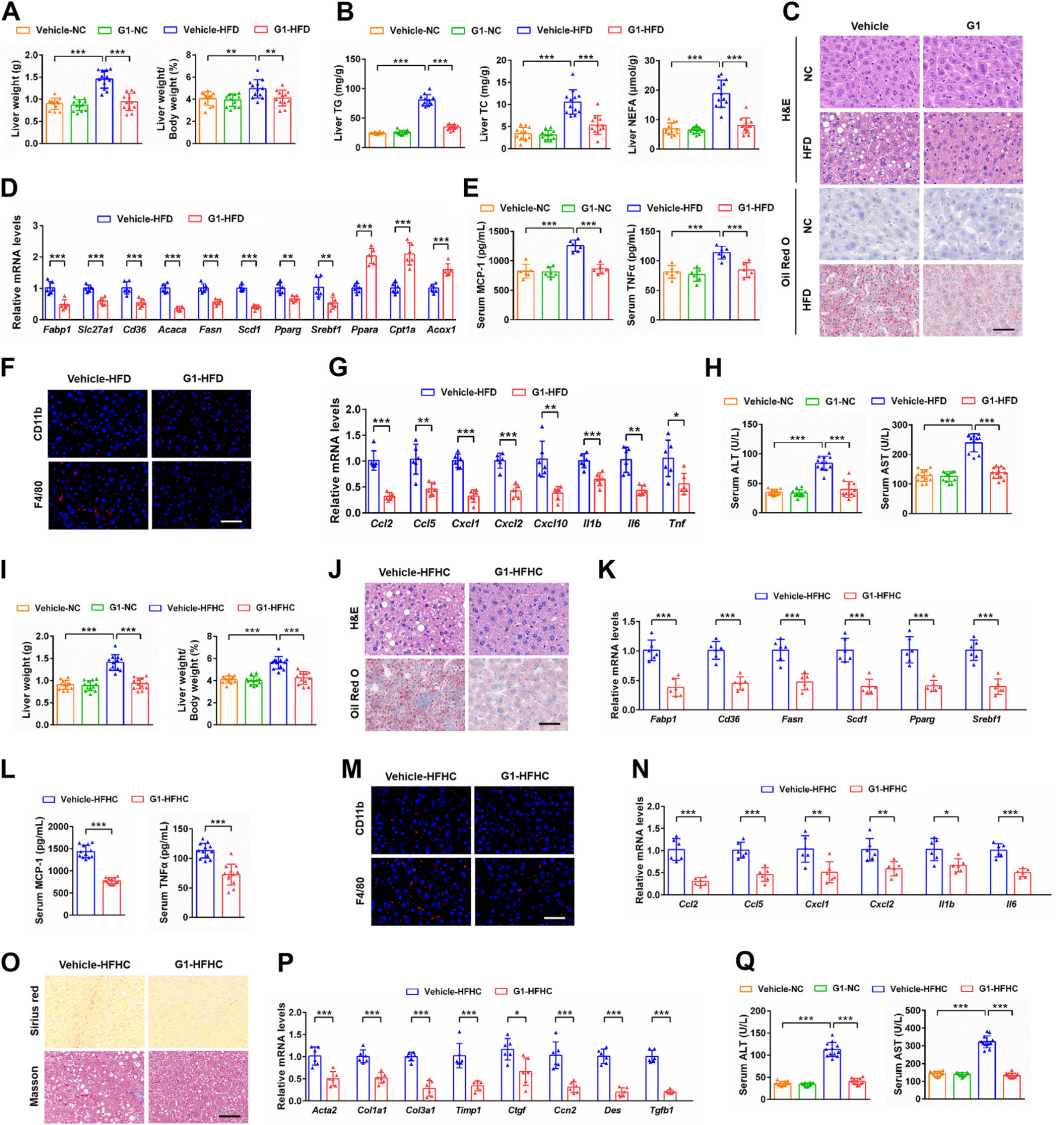
**Activation of GPER1 by G1 mitigates HFD- and HFHC-induced hepatic steatosis, inflammation, or fibrosis in female mice.***A*, liver weight and ratios of liver weight to body weight of normal chow (NC) diet- or high-fat diet (HFD)-fed female mice treated with vehicle or GPER1-specific agonist G1 (5 mg/kg/2 days) for 24 weeks (n = 12 mice/group). *B*, hepatic triglyceride (TG), total cholesterol (TC), and nonesterified fatty acid (NEFA) contents of the female mice in the indicated group (n = 12 mice/group). *C*, representative images of H&E (*upper*) and Oil Red O (*lower*) staining on the liver sections of the female mice in the indicated group (n = 6 mice/group). Scale bar represents 50 μm. *D*, relative mRNA levels of factors related to fatty acid metabolism in the livers of the female mice in the indicated group (n = 6 mice/group). *E*, serum concentration of cytokines MCP-1 and TNFα of the female mice in the indicated group (n = 6 mice/group). *F*, representative images of CD11b (*red*, *upper*) and F4/80 (*red*, *lower*) immunofluorescence staining on the liver sections of the female mice in the indicated group, in which nuclei were stained with DAPI (*blue*) (n = 6 mice/group). Scale bar represents 50 μm. *G*, relative mRNA levels of pro-inflammatory factors in the livers of the female mice in the indicated group (n = 6). *H*, serum alanine aminotransferase (ALT) and aspartate aminotransferase (AST) activities of the female mice in the indicated group (n = 12 mice/group). *I*, liver weight and ratios of liver weight to body weight of GPER1-HKO female mice and their corresponding controls GPER1-Flox female mice after NC or high-fat and high-cholesterol (HFHC) diet consumption for 20 weeks (n = 12 mice/group). *J*, representative images of H&E (*upper*) and Oil Red O (*lower*) staining on the liver sections of the female mice in the indicated group (n = 6). Scale bar represents 50 μm. *K*, relative mRNA levels of factors related to fatty acid metabolism in the livers of the female mice in the indicated group (n = 6 mice/group). *L*, serum concentration of cytokines MCP-1 and TNFα of the female mice in the indicated group (n = 12 mice/group). *M*, representative images of CD11b (*red*, *upper*) and F4/80 (*red*, *lower*) immunofluorescence staining on the liver sections of the female mice in the indicated group, in which nuclei were stained with DAPI (*blue*) (n = 6 mice/group). Scale bar represents 50 μm. *N*, relative mRNA levels of pro-inflammatory factors in the livers of the female mice in the indicated group (n = 6 mice/group). *O*, representative images of Sirius red (*upper*) and Masson (*lower*) staining on the liver sections of the female mice in the indicated group (n = 6 mice/group). Scale bar represents 50 μm. *P*, relative mRNA levels of profibrotic factors in the livers of the female mice in the indicated group (n = 6 mice/group). *Q*, serum ALT and AST activities of the female mice in the indicated group (n = 12 mice/group). In all statistical plots, data are expressed as the mean ± SD. For statistical analysis, a two-tailed Student’s *t* test was used for (*D*), (*G*), (*K*), (*L*), (*N*), and (*P*), and one-way ANOVA with Bonferroni analysis was used for (*A*), (*B*), (*E*), (*H*), (*I*), and (*Q*). The mRNA expression of target genes was normalized to that of Actb. ∗*p* < 0.05, ∗∗*p* < 0.01, ∗∗∗*p* < 0.001, comparison between the indicated groups. GPER1, G protein–coupled estrogen receptor 1; HFD, high fat diet.

Considering that activated GPER1 relieves lipid accumulation and inflammation in hepatocytes under metabolic stress. Therefore, we continued to verify the role of GPER1 in NAFLD pathogenesis by treating female mice with GPER1-specific agonist G1 rather than constructing GPER1 hepatocyte-specific transgenic (GPER1-HTG) mice in response to HFD feeding for 24 weeks (Fig. S8*A*) or HFHC diet feeding for 20 weeks (Fig. S9*A*). In contrast to the phenotypic alterations in HFD-induced NAFLD of GPER1-HKO female mice, the female mice treated with G1 exhibited lower body weight, liver weight, ratios of liver weight to body weight, WAT weight, and ratios of WAT weight to body weight than that of the control mice in response to HFD consumption (Figs. 4*A* and S8, *B* and *C*). Notably, HFD-induced glucose homeostasis impairment and systematic insulin resistance were markedly alleviated by the activation of GPER1 with GPER1-specific agonist G1 in female mice (Fig. S8, *D*–*J*). Also, the female mice treated with G1 significantly attenuated hepatic steatosis, hyperlipidemia, and disorder of lipid metabolism gene expression induced by HFD (Figs. 4, *B*–*D* and S8, *K* and *L*). Furthermore, HFD-induced systematic inflammatory response, hepatic inflammatory cell infiltration, upregulation of pro-inflammatory genes expression, and liver injury were remarkably alleviated in female mice by G1 treatment (Figs. 4, *E*–*H* and S8*M*). In parallel, we found that HFHC-induced body weight gain, hepatic steatosis, inflammation, fibrosis, and liver injury were markedly alleviated in G1-treated female and male mice compared with the control female and male mice (Figs. 4, *I*–*Q*, S9, *B*–*G*, and S10). In general, these results indicate that specific activation of GPER1 by G1 mitigates the progression of HFD- and HFHC-induced NAFLD/NASH in female and male mice.

**Figure 5:**
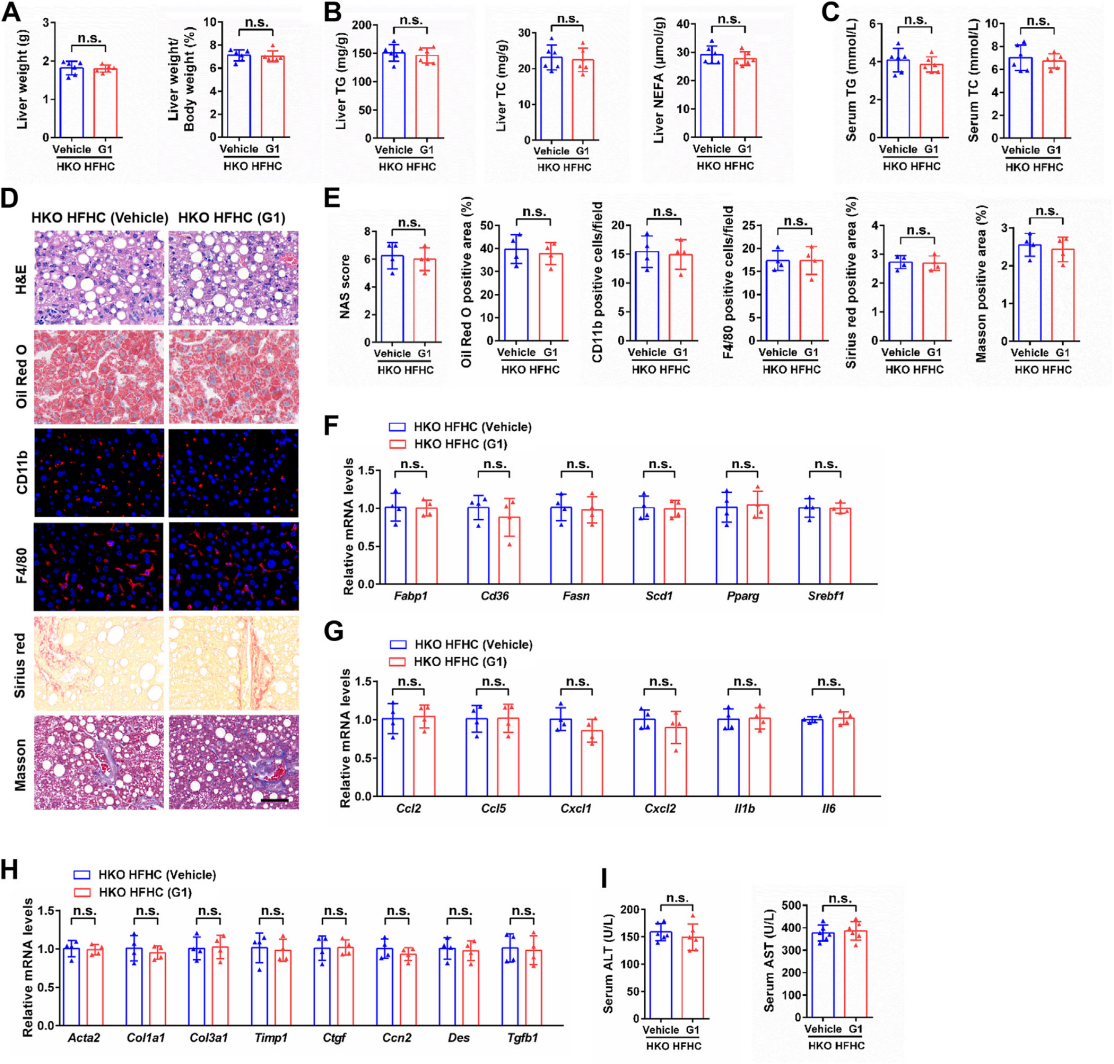
**Lack of protective effect of G1 treatment on NASH induced by HFHC diet in hepatic GPER1-KO female mice.***A*, liver weight and ratios of liver weight to body weight of GPER1-HKO female mice treated with vehicle or GPER1-specific agonist G1 (5 mg/kg/2 days) and fed with high-fat and high-cholesterol (HFHC) diet consumption for 20 weeks (n = 6 mice/group). *B*, hepatic triglyceride (TG), total cholesterol (TC), and nonesterified fatty acid (NEFA) contents of the female mice in the indicated group (n = 6 mice/group). *C*, serum TG and TC contents of the female mice in the indicated group (n = 6 mice/group). *D*, representative images of H&E, Oil Red O, CD11b, F4/80, Sirius red, and Masson staining on the liver sections of the female mice in the indicated group (n = 4 mice/group). Scale bar represents 50 μm. *E*, quantification of H&E, Oil Red O, CD11b, F4/80, Sirius red, and Masson staining in (*D*) (n = 4 mice/group). *F*–*H*, relative mRNA levels of factors related to fatty acid metabolism (*F*), inflammatory response (*G*), and fibrosis (*H*) in the livers of the female mice in the indicated group (n = 4 mice/group). *I*, serum alanine aminotransferase (ALT) and aspartate aminotransferase (AST) activities of the female mice in the indicated group (n = 6 mice/group). In all statistical plots, data are expressed as the mean ± SD and analyzed by a two-tailed Student’s *t* test. The mRNA expression of target genes was normalized to that of Actb. n.s., no significance, *p* ≥ 0.05, comparison between the indicated groups. GPER1, G protein–coupled estrogen receptor 1; NASH, nonalcoholic steatohepatitis.

To verify that hepatic GPER1 was essential to mediate the beneficial effect of G1 on NASH progression, the hepatic GPER1-KO female mice were fed with an HFHC diet and cotreated with vehicle or G1 for 20 weeks. The beneficial role of G1 on the progression of NASH was dispelled in hepatic GPER1-KO female mice, which indicated by no significant differences in hepatic steatosis, inflammation, fibrosis, and liver injury were observed between vehicle and G1-treated GPER1-HKO female mice after HFHC consumption (Fig. 5). Taken together, these “on-target” data confirm that the beneficial role in the progression of NASH is due to direct hepatic GPER1 activation.

### AMPK and its downstream pathways are involved in GPER1-mediated NAFLD/NASH progression

**Figure 6:**
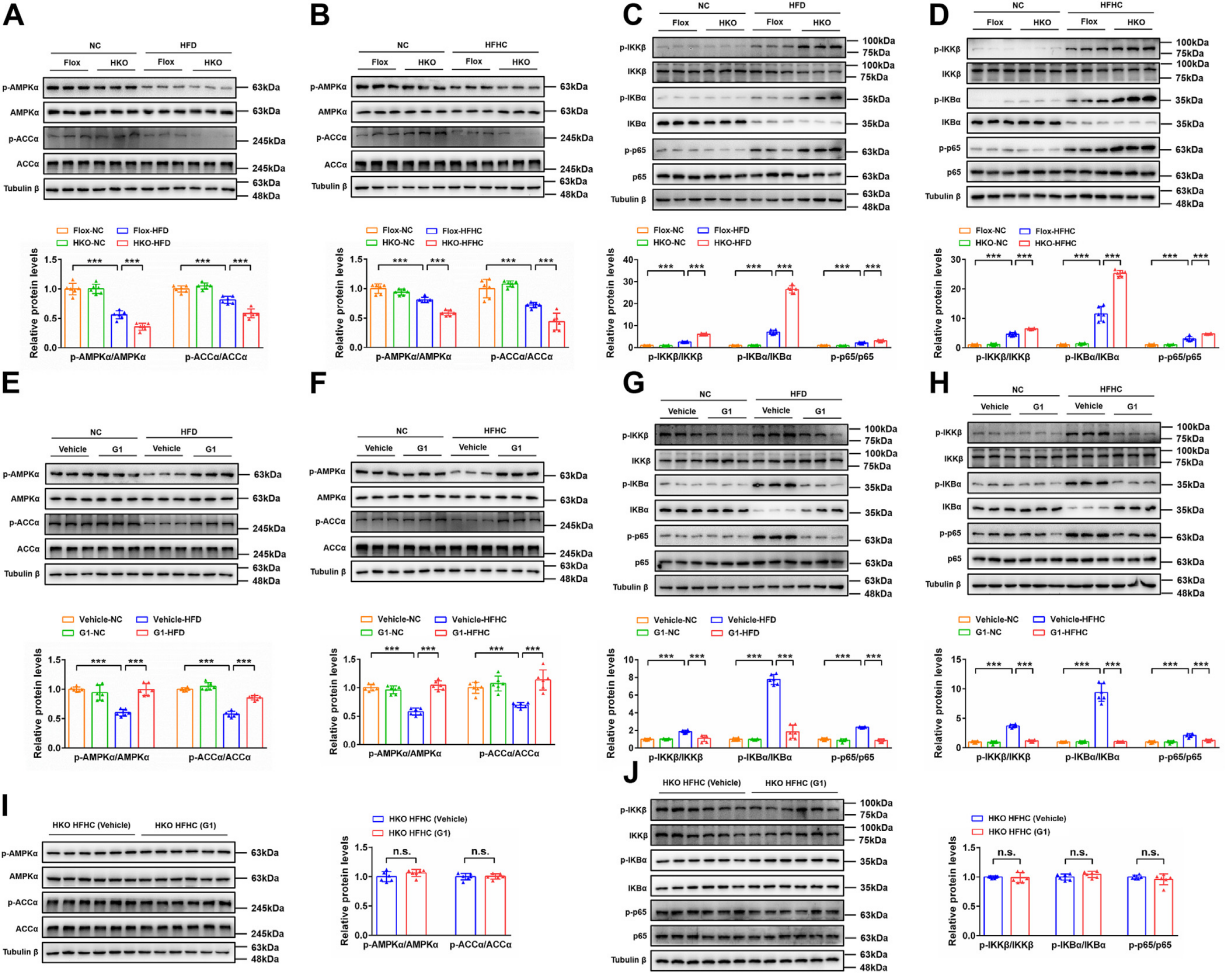
**AMPK and its downstream pathways are involved in GPER1-regulated NAFLD.***A*, immunoblotting analyses of total and phosphorylated AMPKα and ACCα protein levels in the liver tissues of GPER1-HKO female mice and their corresponding controls GPER1-Flox female mice after normal chow (NC) diet or high-fat diet (HFD) consumption for 24 weeks. *B*, immunoblotting analyses of total and phosphorylated AMPKα and ACCα protein levels in the liver tissues of GPER1-HKO female mice and their corresponding controls GPER1-Flox female mice after NC diet or high-fat and high-cholesterol (HFHC) diet consumption for 20 weeks. *C*, immunoblotting analyses of total and phosphorylated IKKβ, IKBα, and p65 protein levels in the liver tissues of GPER1-HKO female mice and their corresponding controls GPER1-Flox female mice after NC or HFD consumption for 24 weeks. *D*, immunoblotting analyses of total and phosphorylated IKKβ, IKBα, and p65 protein levels in the liver tissues of GPER1-HKO female mice and their corresponding controls GPER1-Flox female mice after NC or HFHC consumption for 20 weeks. *E*, immunoblotting analyses of total and phosphorylated AMPKα and ACCα protein levels in the liver tissues of NC diet- or HFD-fed female mice treated with vehicle or GPER1-specific agonist G1 (5 mg/kg/2 days) for 24 weeks. *F*, immunoblotting analyses of total and phosphorylated AMPKα and ACCα protein levels in the liver tissues of NC- or HFHC-diet-fed female mice treated with vehicle or G1 (5 mg/kg/2 days) for 20 weeks. *G*, immunoblotting analyses of total and phosphorylated IKKβ, IKBα, and p65 protein levels in the liver tissues of NC diet or HFD-fed female mice treated with vehicle or G1 (5 mg/kg/2 days) for 24 weeks. *H*, immunoblotting analyses of total and phosphorylated IKKβ, IKBα, and p65 protein levels in the liver tissues of NC- or HFHC-diet-fed female mice treated with vehicle or G1 (5 mg/kg/2 days) for 20 weeks. *I*, immunoblotting analyses of total and phosphorylated AMPKα and ACCα protein levels in the liver tissues of HFHC-diet-fed GPER1-HKO female mice treated with vehicle or G1 (5 mg/kg/2 days) for 20 weeks. *J*, immunoblotting analyses of total and phosphorylated IKKβ, IKBα, and p65 protein levels in the liver tissues of HFHC-diet-fed GPER1-HKO female mice treated with vehicle or G1 (5 mg/kg/2 days) for 20 weeks. In all statistical plots, data are expressed as the mean ± SD. For statistical analysis, a two-tailed Student’s *t* test was used for (*I*) and (*J*), and one-way ANOVA with Bonferroni analysis was used for (*A*–*H*). ∗∗∗*p* < 0.001, comparison between the indicated groups; n.s., no significance, *p* ≥ 0.05, comparison between the indicated groups. For immunoblotting, tubulin β was served as the loading control. AMPK, AMP-activated protein kinase; GPER1, G protein–coupled estrogen receptor 1; HFD, high fat diet; NAFLD, nonalcoholic fatty liver disease.

The protective effect of GPER1 in the pathogenesis of NAFLD/NASH prompted us to further explore its downstream target factors and the potential molecular mechanisms. As an important factor of energy perception and regulation, AMPK has been indicated to be associated with a variety of serious metabolic diseases (29). Accumulated evidence indicated that deletion of liver-specific AMPK exacerbates liver injury and hepatic steatosis in NASH mice (30, 31), and activation of AMPK signaling alleviates the progression of NAFLD/NASH in mice (32, 33, 34). Western blot analysis showed that administration of HFD or HFHC markedly induced the inhibition of AMPK signaling in the livers of female mice, as evidenced by reduced phosphorylation of AMPKα and ACCα (Fig. 6). Numerous studies have demonstrated that AMPK is a negative protein kinase for regulating NF-κB signaling (35), and the present study also found that NF-κB signaling was markedly activated in HFD or HFHC diet group compared with the NC group, as showed by the elevated phosphorylated IKKβ, IKBα, and p65 protein levels in the liver of female mice (Fig. 6). These results suggest that the AMPK signaling was inhibited and the NF-κB signaling was activated in the progression of NAFLD/NASH induced by HFD/HFHC diet. Importantly, AMPK signaling was inhibited but NF-κB signaling was activated in the livers of GPER1-HKO female mice than in the GPER1-Flox control female mice after HFD feeding for 24 weeks or HFHC consumption for 20 weeks (Fig. 6, *A*–*D*). On contrary, activation of GPER1 by G1 largely alleviated HFD or HFHC-induced inhibition of AMPK signaling and activation of NF-κB signaling (Fig. 6, *E*–*H*). However, the beneficial role of G1 on AMPK and downstream NF-κB signaling was completely abolished in GPER1-HKO mice with the consumption of the HFHC diet (Fig. 6, *I* and *J*). Taken together, these data demonstrate that AMPK and its downstream pathways are involved in GPER1-mediated NAFLD/NASH progression.

In parallel with the NAFLD/NASH models *in vivo*, we observed that E2-mediated activation of AMPK signaling and inhibition of NF-κB signaling was completely dispelled in primary hepatocytes from the GPER1-HKO female mice, GPER1 KO L02 cells, and GPER1 KO HepG2 cells under PO stimulation (Fig. S11). Meanwhile, PO-challenged inhibition of AMPK signaling and activation of NF-κB signaling was obviously alleviated by G1 treatment in hepatocytes, including primary mouse hepatocytes from the female mice, L02 cells, and HepG2 cells (Fig. S12). Overall, GPER1 mediates the activation of AMPK signaling and inhibition of NF-κB signaling in NAFLD/NASH models *in vivo* and *in vitro*.

### GPER1 mediates the release of cAMP and activates the PKA-dependent AMPK signaling pathway

**Figure 7:**
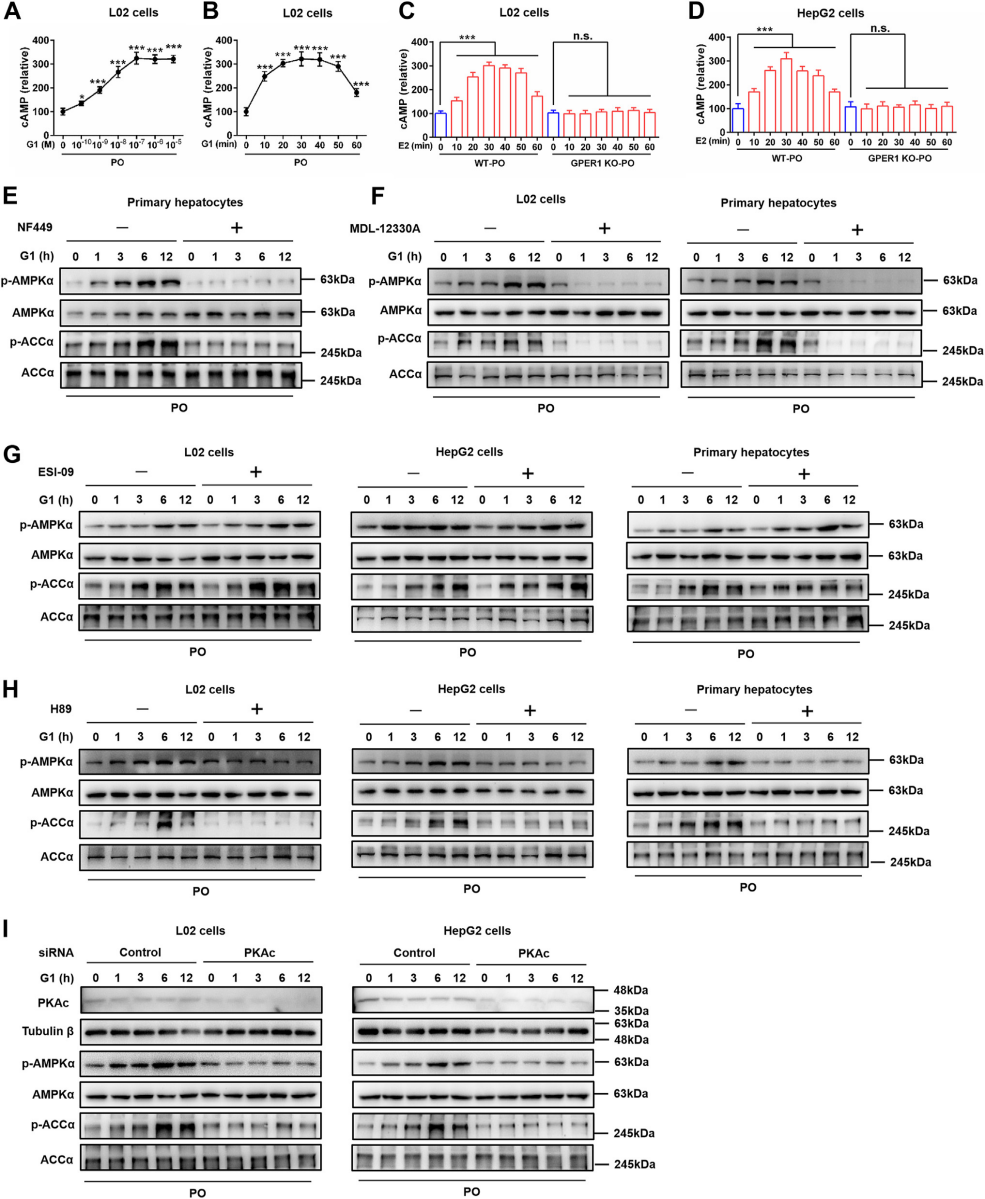
**GPER1 mediates the release of cAMP and activates the AMPK signaling pathway.***A*, cyclic AMP levels in PO (palmitic acid and oil acid mixture)-challenged L02 cells for 30 min after treatment with 10^−10^ to 10^−5^ M GPER1-specific agonist G1 (n = 4). *B*, cyclic AMP levels in PO-challenged L02 cells after treatment with G1 (100 nM) for the indicated times (n = 4). *C*, cyclic AMP levels in WT or GPER1 KO L02 cells challenged by PO and cotreated with vehicle or 17β-estradiol (E2; 10 nM) for the indicated times (n = 3). *D*, cyclic AMP levels in WT or GPER1 KO HepG2 cells were challenged by PO and cotreated with vehicle or E2 for the indicated times (n = 3). *E*, immunoblotting analyses of total and phosphorylated AMPKα and ACCα protein levels in primary hepatocytes that isolate from WT female mice challenged by PO and cotreated with vehicle or G1 in the absence or presence of Gαs inhibitor NF449 (10 μM). *F*, immunoblotting analyses of total and phosphorylated AMPKα and ACCα protein levels in L02 cells (*left*) and primary hepatocytes (*right*) that isolate from WT female mice challenged by PO and cotreated with vehicle or G1 in the absence or presence of AC inhibitor MDL-12330A (20 μM). *G*, immunoblotting analyses of total and phosphorylated AMPKα and ACCα protein levels in L02 cells (*left*), HepG2 cells (*middle*), and primary hepatocytes (*right*) that isolate from WT female mice challenged by PO and cotreated with vehicle or G1 in the absence or presence of EPAC antagonist ESI-09 (10 μM). *H*, immunoblotting analyses of total and phosphorylated AMPKα and ACCα protein levels in L02 cells (*left*), HepG2 cells (*middle*), and primary hepatocytes (*right*) that isolate from WT female mice challenged by PO and cotreated with vehicle or G1 in the absence or presence of PKA inhibitor H89 (10 μM). *I*, immunoblotting analyses of total and phosphorylated AMPKα and ACCα protein levels in L02 cells (*left*) and HepG2 cells (*right*) challenged by PO and cotreated with vehicle or G1 in the absence or presence of PKA catalytic subunit α (PKAc) siRNA. In all statistical plots, data are expressed as the mean ± SD and analyzed by one-way ANOVA with Bonferroni analysis. For (*A*) and (*B*), ∗*p* < 0.05, ∗∗∗*p* < 0.001, G1-PO group *versus* Vehicle-PO group. For (*C*) and (*D*), ∗∗∗*p* < 0.001, comparison between the indicated groups; n.s., no significance, *p* ≥ 0.05, comparison between the indicated groups. AC, adenylyl cyclase; AMPK, AMP-activated protein kinase; EPAC, exchange protein activated by cAMP; GPER1, G protein–coupled estrogen receptor 1; PO, palmitic acid-oleic acid mixture.

Similar to other G-protein-coupled receptors (GPCRs), GPER1 mediates rapid signaling in response to E2, such as inducing cAMP production and subsequently activates the multiple signaling pathways, which have been widely reported (16, 36). cAMP is an important second messenger, and its activation is closely related to AMPK signaling (37). Therefore, we subsequently investigate GPER1-mediated AMPK activation whether associated with the release of cAMP in hepatocytes. In this study, we observed that activation of GPER1 by G1 treatment effectively enhanced cAMP levels in PO-stimulated L02 cells (Fig. 7, *A* and *B*). Moreover, GPER1 deletion completely blocked the E2-mediated increase of cAMP levels in PO-induced L02 cells and HepG2 cells (Fig. 7, *C* and *D*). Furthermore, we found the GLP-1/GCGR agonist cotadutide significantly increased cAMP levels in PO-induced WT L02 cells and HepG2 cells, and no difference was observed in cAMP levels between cotadutide-treated WT hepatocytes and cotadutide-treated GPER1 KO hepatocytes (Fig. S13). These results further indicate that E2-induced cAMP production is attributed to GPER1 activation, and GPER1 is necessary for E2-mediated cAMP production in hepatocytes under metabolic stress. Importantly, we observed that G1-induced activation of the AMPK pathway was completely blocked in isolated primary mouse hepatocytes by pretreatment with the Gαs subunit inhibitor NF449 (Fig. 7*E*). Then, the probability that the enhancement of cAMP levels was responsible for the activation of AMPK by G1 treatment was investigated in L02 cells and primary mouse hepatocytes treatment with G1, either with or without the adenylyl cyclase (AC) inhibitor MDL-12330A. Our data indicated that G1-stimulated AMPK pathway activation was completely blocked by pretreatment with MDL-12330A in primary mouse hepatocytes and L02 cells (Fig. 7*F*). Therefore, these data for the first time to our knowledge suggest that estrogen signaling *via* GPER1, a nonclassical estrogen receptor, can induce cAMP production and directly activate the AMPK pathway in hepatocytes.

cAMP signaling mainly binds to two effectors, including exchange protein activated by cAMP (EPAC) and PKA in multiple cells (37). To explore which effector is involved in mediating the activation of the AMPK pathway by GPER1, the hepatocytes were pretreated with specific inhibitors for EPAC or PKA, respectively. We found that pretreatment with PKA inhibitor H89, rather than EPAC inhibitor ESI-09, blocked the activation of the AMPK pathway mediated by GPER1 in L02 cells, HepG2 cells, and primary mouse hepatocytes (Fig. 7, *G* and *H*). Moreover, GPER1-regulated AMPK activation was completely eliminated in L02 cells and HepG2 cells by transfection with siRNA targeting PKA catalytic subunit (PKAc) (Fig. 7*I*). Taken together, GPER1 mediates the release of cAMP and then activates the PKA-dependent AMPK signaling pathway.

### Inhibition of AMPK abolishes the protective effect of GPER1 activation on NASH progression

To investigate whether AMPK signaling is necessary for the GPER1-mediated protective effects of inflammation and lipid accumulation in hepatocytes, L02 cells were transfected with control or AMPKα KO vectors and then cotreated with GPER1-specific agonist G1 during the PO challenge. The results showed that AMPKα deletion (Fig. S14*A*) completely abrogated the decreases of lipid accumulation and oxidative stress caused by GPER1 activation in PO-stimulated L02 cells (Fig. S14, *B*–*E*). Moreover, the inhibitory effect of G1 on pro-inflammatory mediator expression level was largely eliminated by AMPK knockout in PO-stimulated L02 cells (Fig. S14*F*). Western blot results further confirmed that AMPKα knockout memorably eliminated the inhibitive effect of G1 on the NF-κB pathway (Fig. S14*G*). The inhibitory effect of G1 on lipid accumulation was completely reversed when AMPK signaling was inhibited by the AMPK inhibitor compound C (CC) (Fig. S14*H*), as observed in primary mouse hepatocytes stimulated with PO (Fig. S14*I*). These results demonstrate that activation of AMPK is necessary for the benefits of activated GPER1 on lipid accumulation, oxidative stress, and inflammatory response in hepatocytes.

**Figure 8:**
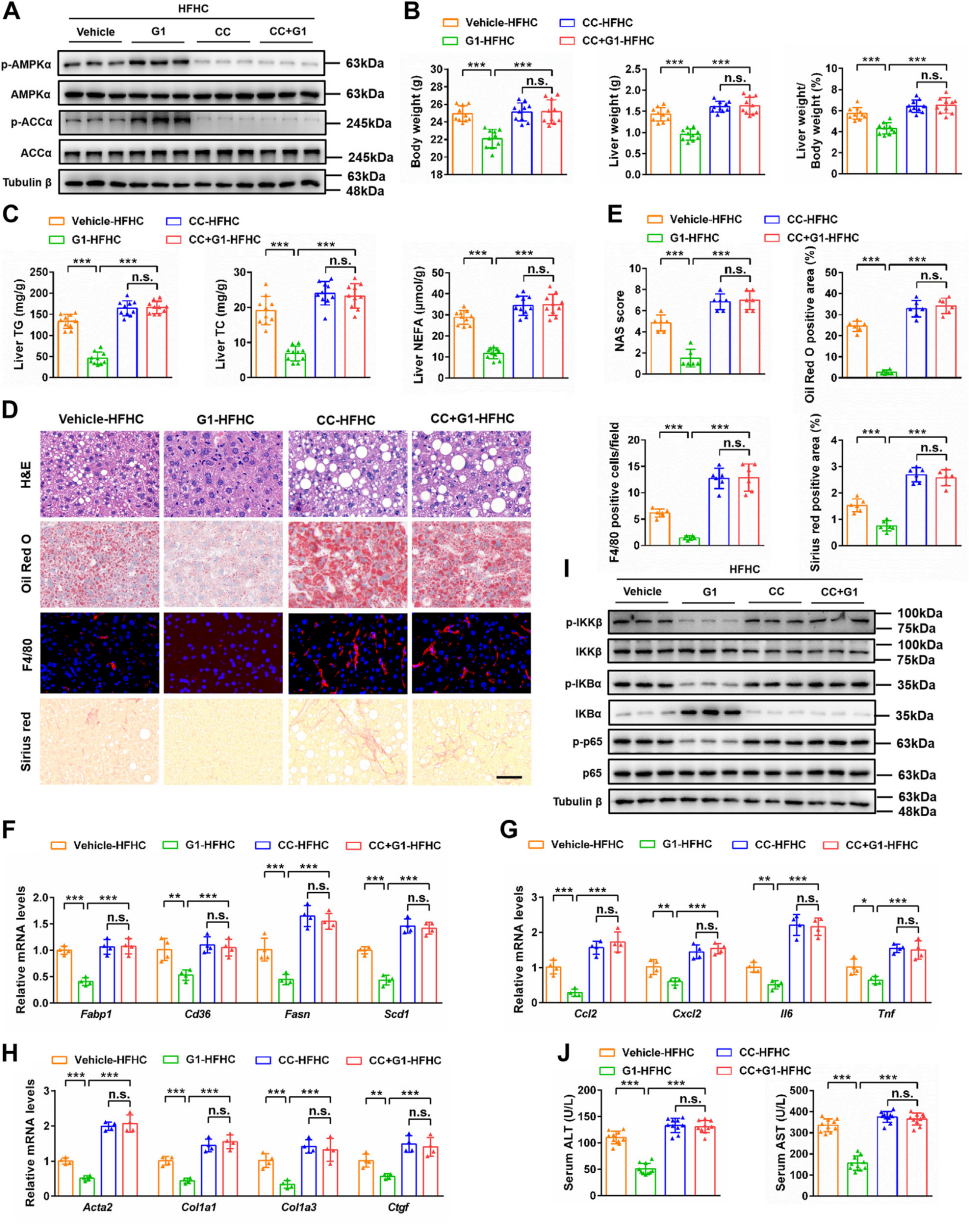
**Inhibition of AMPK abolishes the protective effect of GPER1 activation against HFHC-induced NASH in female mice.***A*, immunoblotting analyses of total and phosphorylated AMPKα and ACCα protein levels in female mice fed a high-fat and high-cholesterol (HFHC) diet and cotreated with vehicle or GPER1-specific agonist G1 (5 mg/kg/2 days) in the absence or presence of compound C (CC, 10 mg/kg/2 days) for 20 weeks (n = 6 mice/group). Tubulin β was served as the loading control. *B*, body weight, liver weight, and ratios of liver weight to body weight of the female mice in the indicated group (n = 10 mice/group). *C*, hepatic triglyceride (TG), total cholesterol (TC), and nonesterified fatty acid (NEFA) contents of the female mice in the indicated group (n = 10 mice/group). *D*, representative images of H&E, Oil Red O, F4/80, and Sirius red staining on the liver sections of the female mice in the indicated group (n = 6 mice/group). Scale bar represents 50 μm. *E*, quantification of H&E, Oil Red O, F4/80, and Sirius red staining in (*D*) (n = 6 mice/group). *F*–*H*, relative mRNA levels of factors related to fatty acid metabolism (*F*), inflammatory response (*G*), and fibrosis (*H*) in the livers of the female mice in the indicated group (n = 4 mice/group). *I*, immunoblotting analyses of total and phosphorylated IKKβ, IKBα, and p65 protein levels in the liver tissues of the female mice in the indicated group (n = 6 mice/group). Tubulin β was served as the loading control. *J*, serum alanine aminotransferase (ALT) and aspartate aminotransferase (AST) activities of the female mice in the indicated group (n = 10 mice/group). In all statistical plots, data are expressed as the mean ± SD and analyzed by one-way ANOVA with Bonferroni analysis. The mRNA expression of target genes was normalized to that of Actb. ∗*p* < 0.05, ∗∗*p* < 0.01, ∗∗∗*p* < 0.001, comparison between the indicated groups; n.s., no significance, *p* ≥ 0.05, comparison between the indicated groups. AMPK, AMP-activated protein kinase; GPER1, G protein–coupled estrogen receptor 1; NASH, nonalcoholic steatohepatitis.

For certified these results *in vitro*, the HFHC diet–fed female mice were pretreated with CC for 1 week and then cotreated with GPER1-specific agonist G1 for another 20 weeks. CC is a selective AMPK inhibitor that has been widely used to inhibit AMPK and evaluate its regulatory effect on NASH in mice (38). We observed that CC treatment markedly abrogated hepatic AMPK activity (Fig. 8*A*) and completely blocked the preventive roles of G1 on the progression of inflammation, hepatic steatosis, and fibrosis in female mice fed with the HFHC diet (Fig. 8, *B*–*J*). Taken together, genetic and pharmacological evidence indicates that activation of AMPK is a crucial downstream effector for the GPER1-mediated anti-NASH.

## Discussion

NASH is a prevalent and multifaceted liver condition characterized by the presence of hepatic steatosis, chronic inflammatory response, hepatic fibrosis, and liver damage, and usually accompanied by the occurrence of obesity, oxidative stress, and insulin resistance. Although extensive and in-depth studies have been conducted on the pathogenesis and progression of NASH, there is a lack of drugs approved for the therapies of NASH worldwide partially due to the unclear pathophysiological mechanisms. In the current study, the benefits and potential mechanisms of GPER1 in NASH progression were investigated, and we certified that the hepatic steatosis, inflammation, fibrosis, or insulin resistance in mouse NAFLD/NASH models were exacerbated by hepatocyte-specific GPER1 knockout but obviously mitigated by hepatic GPER1 activation. Mechanistically, by evaluating the relationship between GPER1 and AMPK signaling, we determined that AMPK as the downstream target molecule of GPER1 and hepatic GPER1 activates AMPK signaling by inducing cyclic AMP release, thereby exerting its protective effect. To our knowledge, our findings are the first to report that GPER1 could be a promising target for anti-NAFLD/NASH.

Accumulating evidence supports the irreplaceable role of estrogen in maintaining lipid and glucose homeostasis (39). Epidemiological studies have consistently shown that estrogen deficiency in menopausal women is linked to a higher incidence of obesity, diabetes, NAFLD, and various other metabolic diseases (40, 41), which were effectively alleviated by estrogen treatment (42). As a result, estrogen replacement therapy has been widely acknowledged as an effective approach for managing metabolic diseases in postmenopausal women. Although the role of nuclear ERs in mediating gene transcription in response to estrogen has been extensively studied, the rapid effects of estrogen prompted the discovery and identification of membrane ERs. GPER1, a membrane ER distinct from nuclear ER, has a wide range of regulatory effects on various metabolic functions through the rapid signaling in response to estrogen (16). Despite research that has extensively explored the nuclear ER as a potential therapeutic target for NAFLD (43), the benefits of GPER1 in NAFLD/NASH progression remain unknown until now. Here, we found that GPER1 prevents NAFLD/NASH by activating AMPK signaling. This is supported by the following evidence. First, GPER1-HKO exacerbates HFD and HFHC diet-challenged hepatic steatosis, inflammation, or fibrosis in female and male mice. Second, specific activation of hepatic GPER1 alleviates the progression of NAFLD/NASH challenged by HFD or HFHC diet in female and male mice. Finally, inhibition of AMPK signaling completely blocked the preventive roles of GPER1 activation on hepatic steatosis, inflammation, and fibrosis in female mice. Hence, our data provide strong evidence indicating the crucial involvement of GPER1 in the progression of NAFLD/NASH in mice.

In this study, we observed that activated GPER1 induced AMPK signaling activation by increasing cAMP release under metabolic stress. cAMP is produced from ATP under the catalysis of AC (37), which is triggered by GPCR, and responds to multiple extracellular stimulations (44). GPER1, a member of the GPCR family, can induce AC activation and increase cAMP release in response to estrogen (16). cAMP is a positive mediator of multiple metabolic regulations, and the identification of AC as the target of GPER1 might explain the beneficial regulatory effect of GPER1 on metabolic disorders. In this study, we certified that the cellular cAMP release and subsequently PKA activation is involved in the activation of AMPK signaling as a result of specific activation of GPER1. AMPK is an important energy perception and regulation factor, which plays a beneficial role in maintaining cellular and metabolic homeostasis (45). Hepatic AMPK activity has been identified as significantly associated with the prevalence of NAFLD, and activation of AMPK prevents NAFLD/NASH progression, including hepatic steatosis, fibrosis, and inflammation (33, 38). Our recent study showed that hepatic AMPK activity was decreased observantly in the NASH model of mice, and activation of AMPK signaling is an effective strategy to block the progress of NASH (22). Our data clarified that AMPK is a crucial downstream molecule for the benefit of GPER1 on NAFLD/NASH progression. The beneficial effect of activated AMPK signaling for anti-NAFLD/NASH can be achieved by regulating the metabolic homeostasis of fatty acids, alleviating oxidative stress and inflammatory response, ameliorating fibrosis, and improving liver injury. Recent studies also indicated that activation of AMPK directly phosphorylates caspase-6 to alleviate liver damage in NASH (30, 34). Moreover, AMPK has been identified exert a crucial role in the activation of the hepatic stellate cell, extracellular matrix secretion, and other stages in liver fibrosis (46). Our data indicated that AMPK activation mediated by GPER1 reduces lipid accumulation by enhancing lipogenesis and reducing lipolysis and ameliorates liver fibrosis by inhibiting the transcription of fibrosis-related factors. Moreover, activation of AMPK signaling effectively suppressed inflammatory responses through suppressing NF-κB signaling. These findings are in line with previous research suggesting that AMPK may serve as a promising target for anti-NASH (30, 47). Taken together, the collective evidence strongly supports the notion that GPER1-mediated the activation of cAMP-PKA–dependent AMPK signaling pathway can effectively impede the progression of NAFLD/NASH.

In conclusion, the novelty of our results suggests that GPER1 prevents the progression of NAFLD/NASH through activation of the cAMP/PKA-dependent AMPK signaling pathway, uncovering GPER1 might be a promising therapeutic target for NAFLD/NASH. However, future translational studies of women and men will ultimately determine the value of G1 in NAFLD patients.

## Experimental procedures

### Key resources table

Detailed information about Key resources table can be found in Supplementary information.

### Experimental model and subject details

#### Mice

The animal experiments described in this study were conducted in accordance with ethical guidelines and approved by the Animal Welfare & Ethical Committee of Nanjing Agricultural University. Female and male C57BL/6J mice, 6 to 8 weeks old, were housed in the pathogen-free barrier facility of the experimental animal center of Nanjing Agricultural University. The mice were maintained in a temperature (23 °C ± 2 deg.) and humidity (50–60%) controlled conditions with a 12 h light/dark cycle and free access to food and drinking water, and animals were divided into different groups according to similar weight before experiment.

For GPER1 activation *in vivo*, mice received GPER1-specific agonist G1 (suspended in 0.5% CMC) by gavage every other day. For AMPK inhibition *in vivo*, mice received AMPK inhibitor compound C (suspended in 0.5% CMC) by gavage every other day. Bilateral OVX was performed as a surgical model of menopause.

### NAFLD, NASH, and metabolic disorders mice models

#### HFD-induced NAFLD model

To establish the NAFLD model, female mice were fed with the HFD (60% kcal from fat, 20% kcal from protein, 20% kcal from carbohydrates; XTHF60, XIETONG Pharmaceutical Bioengineering) for 24 weeks. The mice were fed with a NC diet (10% kcal from fat, 20% kcal from protein, 70% kcal from carbohydrate; XIETONG Pharmaceutical Bioengineering) as the corresponding controls.

#### HFHC diet-induced NASH model

The NASH model was established by feeding the female and male mice a HFHC diet (40% kcal from fat, 20% kcal from protein, 40% kcal from carbohydrate, and 1.8% cholesterol; XT301, XIETONG Pharmaceutical Bioengineering) for 20 and 16 weeks, respectively. The mice fed with the NC diet (10% kcal from fat, 20% kcal from protein, 70% kcal from carbohydrate; XIETONG Pharmaceutical Bioengineering) as the corresponding controls.

#### Metabolic disorders model

The female mice were surgically removed from bilateral ovaries and fed with the NC diet for 20 weeks to establish an estrogen deficiency–induced obesity and metabolic disorders model of postmenopausal women, and the sham-operated mice were fed with the NC diet as the corresponding controls.

### Mouse primary hepatocytes

Primary mouse hepatocytes were obtained from 6- to 8-week-old female mice using liver perfusion as previously described (48). Briefly, the mice were anesthetized with 2% pentobarbital sodium and then perfused with Hanks’ Balanced Salt Solution at 37 °C for approximately 5 to 10 min, followed by liver digest medium containing 0.05% type IV collagenase at 37 °C for another 5 to 10 min through the portal vein. The liver was then collected and filtered through a 100 mm steel mesh. After centrifugation at 50*g* for 1 min, the hepatocytes were cultured in Dulbecco’s modified Eagle’s medium with 10% fetal bovine serum (FBS) and 1% penicillin-streptomycin in a 37 °C and 5% CO_2_ cell incubator. An *in vitro* model of hepatic steatosis and inflammation of primary hepatocytes was established by treating hepatocytes with 0.2 mM PA and 0.4 mM OA mixture (0.6 mM PO; dissolved in 0.6% fatty acid–free bovine serum albumin (BSA)) for 12 h. The primary hepatocytes were stimulated with 0.6% fatty acid–free BSA as the corresponding controls.

### Cell lines

The HepG2 cell lines (human liver carcinoma cell) were purchased from the Cell Bank of Type Culture Collection of the Chinese Academy of Sciences and maintained in Dulbecco’s modified Eagle’s medium with 10% FBS and 1% penicillin-streptomycin. The L02 cell lines (human normal hepatocyte) were purchased from the China Center for Type Culture Collection and cultured in Roswell Park Memorial Institute 1640 medium with 10% FBS and 1% penicillin-streptomycin. All the cell lines were checked for *mycoplasma* contamination once per month and placed in a 37 °C and 5% CO_2_ cell incubator. An *in vitro* model of hepatic steatosis and inflammation was established by treating cells with 0.5 mM PA and 1 mM OA mixture (1.5 mM PO; dissolved in 1.5% fatty acid-free BSA) for 12 h. Cells were stimulated with 1.5% fatty acid-free BSA as the corresponding controls.

### Generation of genetically modified mice

Hepatocyte-specific GPER1 knockout (GPER1-HKO) mice based on the C57BL/6J background were generated using the Cre/loxP recombination. The GPER1^flox/flox^ (GPER1-Flox) mice were established and generated from the Cyagen. Detailed knockout strategy description of GPER1^flox/flox^ mice can be found on the website: https://www.cyagen.com/cn/zh-cn/sperm-bank-cn/S-CKO-16576. To generate GPER1-HKO mice, GPER1^flox/flox^ mice were crossed with albumin-Cre mice (Jackson Laboratory) with the help of Cyagen. GPER1^flox/flox^ mice were bred with GPER1^flox/flox^ Cre/+ mice to generate GPER1-Flox (littermate controls) and GPER1-HKO mice. The genotyping primers of flox and Cre are listed in Table S1.

### Organ indexes analyses

The organs, including the heart, liver, spleen, lung, and kidney, were thoroughly separated and weighed. The organ indexes were calculated as the ratios of organ weight to body weight. WAT is the sum of perirenal fat, subcutaneous fat, mesenteric fat, and epididymal fat in male mice and the sum of perirenal fat, subcutaneous fat, mesenteric fat, and uterine fat in female mice.

### Metabolic parameter analysis

The levels of fasting blood glucose and fasting serum insulin were measured in 12-h-fasted mice by using the ACCU-Chek Active (Roche Diagnostics) and insulin ELISA kit (Shanghai Hengyuan Biotechnology Co, Ltd) according to the manufacturer's instructions, respectively. HOMA-IR values were calculated. ITTs and GTTs were performed in mice by intraperitoneal injection of insulin (0.75 IU/kg body weight; Thermo Fisher Scientific) and glucose (1 g/kg body weight; Sigma-Aldrich), and then the blood glucose levels at 0 (baseline), 15, 30, 60, 90, and 120 min after injection were monitored by using the ACCU-Chek Active; the areas under the curves were calculated and indicated the differences between different groups.

### Hepatic lipids analyses

The hepatic TG, TC, and non-esterified fatty acid contents were measured using the commercial kits (Nanjing Jiancheng Biotechnology Institution) following the manufacturer’s instructions.

### Mouse serum assays

The content of serum TG and TC, and the activity of ALT and AST were measured using an Automatic Blood Biochemical Analyzer (Beckman Coulter). The serum TNFα and MCP-1 (CCL2) levels were measured using commercial ELISA kits (Shanghai Hengyuan Biological Technology Co, Ltd).

### Immunofluorescence staining

Immunofluorescence staining was performed as our previously described (22). Briefly, liver tissue samples of mice were collected and fixed with 4% paraformaldehyde for at least 12 h at room temperature followed by paraffin embedding. After dewaxing and antigen repair steps, sections were blocked with 5% BSA for 30 min at room temperature and then stained with polyclonal antibodies recognizing CD11b and F4/80 overnight. The sections were washed three times with PBS for 5 min each and then incubated with Cy3-labeled fluorescent secondary antibody for 50 min at room temperature. Then, the samples were stained with DAPI staining solution for another 10 min and then washed three times with PBS for 5 min each. The sections were photographed by a fluorescence microscope (Nikon) and quantified with the software of Image-Pro plus 6.0.

### Histopathological analysis

Liver tissue samples of mice were fixed with 4% paraformaldehyde for at least 12 h at room temperature. Optimum cutting temperature–embedded frozen and paraffin-embedded liver sections were performed for Oil Red O, H&E, Masson, or Sirius red staining. Lipid droplet accumulation was assessed by Oil Red O staining. Liver fibrosis was detected by Masson and Sirius red staining. H&E staining was performed to assess the NAFLD activity score. The sections were photographed by a fluorescence microscope (Nikon) and quantified with the software of Image-Pro plus 6.0.

### Construction and transfection of overexpression vector

The plasmids encoding human full-length GPER1 were constructed by cloning the coding regions of human GPER1 cDNA into the pEX-3(pGCMV/MCS/Neo) plasmid vector. The construction of GPER1 overexpression plasmid vector was obtained from GenePharma and transfected into L02 cells or HepG2 cells using Lipofectamine 3000 reagent according to the manufacturer's instructions.

### siRNA transfection

L02 cells or HepG2 cells were transfected with siRNA targeting human PRKACA (PKAc) and ERα (GenePharma, Shanghai) using Lipofectamine 3000 reagent according to the manufacturer's instructions. The sequences of siRNAs targeting PKAc were as follows (5′-3′): GGAACCACUAUGCCAUGAATT (sense), UUCAUGGCAUAGUGGUUCCTT (antisense). The sequences of siRNAs targeting ERα were as follows (5′-3′): GCCAAAUUCAGAUAAUCGATT (sense), UCGAUUAUCUGAAUUUGGCTT (antisense). After transfection for 36 h in medium, the L02 cells or HepG2 cells were treated as described above.

### Generation of stable KO cell lines

L02 and HepG2 cell lines deficient for specific target genes were established using the CRISPR/Cas9 system. Three sgRNAs targeting genes involved in human GPER1, PRKAA1, and PRKAA2 were designed and cloned into the pCas-Puro-U6 plasmid vector. The recombinant plasmid was generated and obtained from Corues Biotechnology. Primer sequences used to construct sgRNA expression plasmids and genotypes are listed in Table S2. To generate GPER1 and AMPKα1/α2 double-KO cell lines, 60% confluent L02 hepatocytes or HepG2 cells in 24-well plates were transfected with recombinant plasmid targeting GPER1 or PRKAA1/PRKAA2 by using Lipofectamine 3000 reagent multiple times according to the manufacturer's instructions. After being transfected for 48 h, positive candidates were selected by adding 3 μg/ml puromycin. Single cells were cultured in 96-well plates separately to facilitate the growth of cell clones for 2 weeks and then transferred to 24-well plates for another 2 weeks. Cell-positive clones were screened by Western blot.

### Cellular Nile Red staining

For the measurement of lipid droplet accumulation, cells were stained with 0.05 mg/ml Nile Red staining solution at 37 °C for about 20 min and then washed three times with PBS to remove unbound Nile Red staining solution. Subsequently, DAPI staining was used to illustrate the location of the cell nucleus. The lipid droplets emit red fluorescence and the cell nucleus shows blue fluorescence, and cells were photographed using laser scanning confocal microscopy (Zeiss).

### Cellular lipid analysis

Cells were broken in an ice water bath using an ultrasonic crusher, and then the supernatants were collected by centrifugation at 12,000 rpm for 20 min. The cellular TG and TC contents were measured using commercial kits (Nanjing Jiancheng Biotechnology Institution) according to the manufacturer’s instructions.

### Intracellular reactive oxygen species and mitochondrial reactive oxygen species analysis

To analyze intracellular reactive oxygen species (ROS) levels in hepatocytes (HepG2 cells and L02 cells), the cells were exposed to a 10 μM DCFH-DA probe at 37 °C for 20 to 30 min. After that, the cells were washed twice with PBS to eliminate any unbound fluorescent probe. The ROS were visualized as green fluorescence and captured using a fluorescence microscope (Thermo Fisher Scientific). To determine the production of mitochondrial ROS in hepatocytes, the cells were treated with 2.5 μM MitoSOX Red probe for 20 to 30 min. Subsequently, the cells were washed twice with PBS to remove any excess fluorescent probe. The mitochondrial ROS were visualized as red fluorescence and captured using a fluorescence microscope (Thermo Fisher Scientific).

### Cellular cAMP analysis

Cell lysis was achieved by subjecting the cells to an ultrasonic crusher in an ice water bath, and then the supernatants were obtained by centrifugation at 12,000 rpm for 20 min. The cellular cAMP levels were determined using commercially available ELISA kits (Shanghai Hengyuan Biological Technology Co, Ltd).

### Western blot analysis

Western blot analysis was performed following a previously described protocol (49). Briefly, protein extraction was done from liver tissues or cultured hepatocytes using RIPA lysis buffer with 1% protease and phosphatase inhibitor. The lysate was centrifuged at 4 °C, and the protein concentration in the supernatant was quantified using a BCA Protein Assay Kit (Beyotime Biotechnology). The proteins were then separated by 10% SDS-PAGE gels and transferred onto PVDF membranes. The membranes were blocked with 5% skim milk, followed by incubation with specific primary antibodies overnight at 4 °C. After that, the membranes were incubated with secondary antibodies conjugated with horseradish peroxidase for 2 h at room temperature. Protein expression levels were visualized using an ECL reagent substrate. Tubulin β was used as a loading control. The quantification of protein expression levels was performed using Image J Software (National Institutes of Health).

### RNA extraction and real-time qPCR

Total RNA was extracted from the liver tissues of mice or cultured hepatocytes and then reverse transcribed into cDNA. For PCR amplification, SYBR Green PCR Master Mix was utilized. The mRNA expression levels of target genes were normalized to Actb and calculated using the 2^−ΔΔCT^ method. The primer sequences of RT-qPCR are provided in Table S3 and were synthesized by Tsingke Biotechnology Co, Ltd.

### Statistical analysis

Statistical analysis was performed using SPSS 20.0 software (IBM Corporation). The data were expressed as mean ± SD. Two-tailed Student's *t* test was used to compare the two groups, and one-way ANOVA followed by Tukey's *post hoc* test for data meeting homogeneity of variance or with Tamhane's T2 analysis for data of heteroscedasticity was applied for comparisons among multiple groups (more than two groups). A *p*-value of less than 0.05 was considered statistically significant.

## Data availability

All data associated with this study are present in the paper or the Supporting information.

## Supporting information

This article contains supporting information.

## Conflict of interest

All authors declare that they have no conflicts of interests with the contents of this article.
