# Supplementary material for: G protein–coupled estrogen receptor 1 ameliorates nonalcoholic steatohepatitis through targeting AMPK-dependent signaling
Source: J Biol Chem. 2024 Jan 20;300(3):105661. doi: 10.1016/j.jbc.2024.105661 (PMC10876613; doi:10.1016/j.jbc.2024.105661)
Supplement: Supplemental Figures and Tables [file mmc1.docx]

**Supplementary information**

**Key resources table**

| Reagent or resource | Source | Identifier |
| --- | --- | --- |
| Antibodies |  |  |
| Anti-phospho-AMPKα (Thr172), dilution: 1/1000 | Cell Signaling Technology | Cat#2535 |
| Anti-AMPKα, dilution: 1/1000 | Cell Signaling Technology | Cat#5831 |
| Anti-phospho-Acetyl-CoA Carboxylase (Ser79), dilution: 1/1000 | Cell Signaling Technology | Cat#3661 |
| Anti-Acetyl-CoA Carboxylase, dilution: 1/1000 | Cell Signaling Technology | Cat#3662 |
| Anti-phospho-NF-κB p65 (Ser536), dilution: 1/1000 | Cell Signaling Technology | Cat#3033 |
| Anti-NF-κB p65, dilution: 1/1000 | Cell Signaling Technology | Cat#8242 |
| Anti-phospho-AKT, dilution: 1/1000 | Cell Signaling Technology | Cat#9271 |
| Anti-AKT, dilution: 1/1000 | Cell Signaling Technology | Cat#9272 |
| Anti-IKKβ, dilution: 1/1000 | Cell Signaling Technology | Cat#2678 |
| Anti-Estrogen Receptor α, dilution: 1/1000 | Cell Signaling Technology | Cat#13258 |
| Anti-Tubulin β, dilution: 1/10000 | Bioworld Technology | Cat#AP0064 |
| Anti-phospho-GSK3β, dilution: 1/1000 | Bioworld Technology | Cat#BS4084 |
| Anti-phospho-IRS1 (Tyr612), dilution: 1/1000 | Bioworld Technology | Cat#BS4257 |
| Anti-IRS1, dilution: 1/1000 | Bioworld Technology | Cat#BS3590 |
| Anti-phospho-IKKβ (Tyr199), dilution: 1/1000 | Abcam | Cat#ab59195 |
| Anti-IkBα, dilution: 1/1000 | Proteintech Group | Cat#10268-1-AP |
| Anti-PRKACA, dilution: 1/5000 | Proteintech Group | Cat#27398-1-AP |
| Anti-GSK3β, dilution: 1/5000 | Proteintech Group | Cat#67329-1-Ig |
| Anti-phospho-IKBα (Ser32/Ser36), dilution: 1/1000 | Affinity Biosciences | Cat#AF2002 |
| Anti-GPER1, dilution: 1/2000 | Affinity Biosciences | Cat#DF2737 |
| Anti-F4/80, dilution: 1/1000 | Servicebio | Cat#GB11027 |
| Anti-CD11b, dilution: 1/1000 | Servicebio | Cat#GB11058 |
| Goat anti-Rabbit IgG-HRP, dilution: 1/10000 | Proteintech Group | Cat#SA00001-2 |
| Goat anti-mouse IgG-HRP, dilution: 1/10000 | Proteintech Group | Cat# SA00001-1 |
| Cy3 conjugated Goat Anti-Rabbit IgG (H+L), dilution: 1/10000 | Servicebio | Cat#GB21303 |
| Chemicals and reagents |  |  |
| 17β-estradiol sustained release tablet | Innovative Research of America | Cat#NE-121 |
| 17β-estradiol (E2) | Sigma-Aldrich | Cat#E1024 |
| G15 | Selleck | Cat#S6651 |
| ESI-09 | Selleck | Cat#S7499 |
| H89 | Selleck | Cat#S1582 |
| PHTPP | Selleck | Cat#S8686 |
| G1 | APExBIO | Cat#B5455 |
| Compound C | APExBIO | Cat#B3252 |
| MDL-12330A | APExBIO | Cat#B6739 |
| NF449 | APExBIO | Cat#B6716 |
| Palmitic acid | Sigma-Aldrich | Cat#P0500 |
| Oleic acid | Sigma Aldrich | Cat#O1008 |
| Puromycin | Sigma Aldrich | Cat#P9620 |
| Insulin | Thermo | Cat#12585-014 |
| Nile Red | Sigma-Aldrich | Cat#19123 |
| Reactive oxygen species (DCFH-DA probe) assay kit | Beyotime Biotechnology | Cat#S0033 |
| MitoSOX™ red mitochondrial superoxide indicator | Thermo | Cat#M36008 |
| BSA, essentially fatty acid free | Equitech-Bio | Cat#BAH66-0100 |
| DMSO | Sigma-Aldrich | Cat#D2650 |
| Roswell Park Memorial Institute (RPMI) 1640 | Hyclone | Cat#SH30027 |
| Dulbecco’s modiﬁed Eagle’s medium (DMEM) | Hyclone | Cat#SH30022 |
| Fetal bovine serum (FBS) | Gibco | Cat#10270106 |
| 0.25% Trypsin | Gibco | Cat#15140-122 |
| Penicillin/streptomycin | Beyotime Biotechnology | Cat#C0222 |
| DAPI | Invitrogen | Cat#S3693 |
| Lipofectamine 3000 reagent | Thermo | Cat#L3000015 |
| SYBR Green PCR Master Mix | Roche | Cat#04887352001 |
| Trizol | Sigma-Aldrich | Cat#T9424 |
| HiScript® reverse transcription kit | Vazyme Biotech Co., Ltd | Cat#R123-01 |
| BCA protein assay kit | Beyotime Biotechnology | Cat#P0011 |
| Sirius red | Servicebio | Cat#GP1138 |
| Hematoxylin & Eosin (H&E) | Servicebio | Cat#G1005 |
| Masson | Servicebio | Cat#G1006 |
| Oil Red O | Servicebio | Cat#G1016 |
| PAS | Servicebio | Cat#GP1039 |
| Triglyceride (TG) assay kit | Jiancheng Biotechnology | Cat#A110-1 |
| Total cholesterol (TC) assay kit | Jiancheng Biotechnology | Cat#A111-1 |
| NEFA assay kit | Jiancheng Biotechnology | Cat#A042-2-1 |
| Triton X-100 | Sigma Aldrich | Cat#T8787 |
| RIPA lysis buffer | Beyotime Biotechnology | Cat#P0013B |
| Protease and phosphatase inhibitor | Beyotime Biotechnology | Cat#P1045 |
| PMSF | Beyotime Biotechnology | Cat#ST506 |
| Immobilon-P PVDF Membrane | Millipore | Cat#IPVH00010 |
| SuperSignal™ West Pico PLUS | Thermo | Cat#34580 |
| Experimental models: cell lines |  |  |
| Mouse: primary hepatocytes | This paper | N/A |
| Human: L02 cells | China Center for Type Culture Collection | N/A |
| Human: HepG2 cells | Cell Bank of Type Culture Collection of the Chinese Academy of Sciences | Cat#SCSP-510 |
| Experimental models: animal |  |  |
| Mouse: albumin-Cre transgenic | Jackson Laboratory | Cat#003574 |
| Mouse: hepatocyte-specific GPER1 knockout mice (GPER1-HKO) | Cyagen | Cat#S-CKO-16576 |
| Mouse: GPER1 knockout mice (GPER1^-/-^) | Cyagen | Cat#S-KO-14957 |
| Mouse: C57BL/6J (WT) | Beijing Vital River | N/A |
| Oligonucleotides |  |  |
| Primers for genotyping the hepatocyte-specific GPER1 knockout mice, see **Table S1** | This paper | N/A |
| Primers for generating gene knockout L02 cells and HepG2 cells, see **Table S2** | Corues Biotechnology | N/A |
| Primers for RT-qPCR, see **Table S3** | This paper | N/A |

**Table S1. Genotyping primers for knockout mice construction.**

| Gene | Genotyping primers (5'-3') |
| --- | --- |
| Flox | R: CTCGACAAACAGTAGTTTTCCTCA  F: ATAGACGTGGAGTGTTCCTTTATGT |
| Alb-cre | R: GAAGCAGAAGCTTAGGAAGATGG  F: TTGGCCCCTTACCATAACTG |

**Table S2. Oligonucleotide sequences for the generation of gene knockout L02 cell and HepG2 cell lines.**

| Gene | | Target sequences | Genotyping primers (5'-3') |
| --- | --- | --- | --- |
| GPER1 | sg-1: caccgTTGGCAAACATGAATTGACT  sg-2: aaacAGTCAATTCATGTTTGCCAAc | | R: CCAGGTACCCAGAGAGTGAGC  F: TCCAGATGAGGCCACAGCTCAG |
| PRKAA1 | sg-1: caccgAATCTGCTGAAAGAGCCGCC  sg-2: aaacGGCGGCTCTTTCAGCAGATTc | | F: AGCTGGGATTACAGCTGTGTAC  R: ATGTGGGCATTTGGAGGTCAAG |
| PRKAA2 | sg-1: caccGATGTGACTTCCCAAGCCCG  sg-2: aaacCGGGCTTGGGAAGTCACATC | | F: TGTTGCCCAGGCTGGAGCG  R: CCTTAGGAGAAAATGCAGATTGAATGG |

**Table S3. Primer sequences of RT-qPCR**

| Target gene | Primer sequences | Orientation |
| --- | --- | --- |
| Mouse *Actb* | 5'-GTGACGTTGACATCCGTAAAGA-3'  5'-GCCGGACTCATCGTACTCC-3' | Forward  Reverse |
| Mouse *Fabp1* | 5'-TGGTCCGCAATGAGTTCACCCT-3'  5'-CCAGCTTGACGACTGCCTTGACTT-3' | Forward  Reverse |
| Mouse *Slc27a1* | 5'-TGCACAGCAGGTACTACCGCAT-3'  5'-TGCGCAGTACCACCGTCAAC-3' | Forward  Reverse |
| Mouse *Cd36* | 5'-GACTGGGACCATTGGTGATGA-3'  5'-AAGGCCATCTCTACCATGCC-3' | Forward  Reverse |
| Mouse *Acaca* | 5'-GGCCAGTGCTATGCTGAGAT-3'  5'-AGGGTCAAGTGCTGCTCCA-3' | Forward  Reverse |
| Mouse *Fasn* | 5'-CTGCGGAAACTTCAGGAAATG-3'  5'-GGTTCGGAATGCTATCCAGG-3' | Forward  Reverse |
| Mouse *Scd1* | 5'-TCTTCCTTATCATTGCCAACACCA-3'  5'-GCGTTGAGCACCAGAGTGTATCG-3' | Forward  Reverse |
| Mouse *Pparg* | 5'-ATTCTGGCCCACCAACTTCGG-3'  5'-TGGAAGCCTGATGCTTTATCCCCA-3' | Forward  Reverse |
| Mouse *Srebf1* | 5'-CACTTCTGGAGACATCGCAAAC-3'  5'-ATGGTAGACAACAGCCGCATC-3' | Forward  Reverse |
| Mouse *Ppara* | 5'-TATTCGGCTGAAGCTGGTGTAC-3'  5'-CTGGCATTTGTTCCGGTTCT-3' | Forward  Reverse |
| Mouse *Cpt1a* | 5'-AGGACCCTGAGGCATCTATT-3'  5'-ATGACCTCCTGGCATTCTCC-3' | Forward  Reverse |
| Mouse *acta2* | 5'-CTGACAGAGGCACCACTGAA-3'  5'-GAAGGAATAGCCACGCTCAG-3' | Forward  Reverse |
| Mouse *Col1a1* | 5'-TCCTCCAGGGATCCAACGA-3'  5'-GGCAGGCGGGAGGTCTT-3' | Forward  Reverse |
| Mouse *Col3a1* | 5'-CTGGTCAGCCTGGAGATAAG-3'  5'-ACCAGGACTACCACGTTCAC-3' | Forward  Reverse |
| Mouse *Timp1* | 5'-GGCATCTGGCATCCTCTTGT-3'  5'-GCTGGTATAAGGTGGTCTCGT-3' | Forward  Reverse |
| Mouse *Ctgf* | 5'-TGACCCCTGCGACCCACA-3'  5'-TACACCGACCCACCGAAGACACAG-3' | Forward  Reverse |
| Mouse *Ccn2* | 5'-AGAACTGTGTACGGAGCGTG-3'  5'-GTGCACCATCTTTGGCAGTG-3' | Forward  Reverse |
| Mouse *Des* | 5'-TCAGCGAGGCTACACAGCAACA-3'  5'-GGTTGGGCAGCATGAAGACCACAA-3' | Forward  Reverse |
| Mouse *Tgfb1* | 5'-ATTTGGAGCCTGGACACACA-3'  5'-GAGCGCACAATCATGTTGGA-3' | Forward  Reverse |
| Mouse *Ccl2* | 5'-TACAAGAGGATCACCAGCAGC-3'  5'-ACCTTAGGGCAGATGCAGTT-3' | Forward  Reverse |
| Mouse *Ccl5* | 5'-TGCTGCTTTGCCTACCTCTC-3'  5'-TCTTCTCTGGGTTGGCACAC-3' | Forward  Reverse |
| Mouse *Cxcl1* | 5'-TGTGCGAAAAGAAGTGCAG-3'  5'-TACAAACACAGCCTCCCACA-3' | Forward  Reverse |
| Mouse *Cxcl2* | 5'-GCGCCCAGACAGAAGTCATA-3'  5'-CAGTTAGCCTTGCCTTTGTTCA-3' | Forward  Reverse |
| Mouse *Cxcl10* | 5'-ATGACGGGCCAGTGAGAATG-3'  5'-ATGATCTCAACACGTGGGCA-3' | Forward  Reverse |
| Mouse *Tnf* | 5'-CTGTGAAGGGAATGGGTGTT-3'  5'-CAGGTCACTGTCCCAGCATC-3' | Forward  Reverse |
| Mouse *Il6* | 5'-AGTTGTGCAATGGCAATTCTGA-3'  5'-AGGACTCTGGCTTTGTCTTTCT-3' | Forward  Reverse |
| Mouse *Il1β* | 5'-ACCTGTGTCTTTCCCGTGG-3'  5'-TCATCTCGGAGCCTGTAGTG-3' | Forward  Reverse |
| *Mouse Gper1* | 5'-CGCAGTGGTCCTTGTTT-3'  5'-GGTAGGCGTGACGGAAA-3' | Forward  Reverse |
| Human *Actb* | 5'-CATGTACGTTGCTATCCAGGC-3'  5'-CTCCTTAATGTCACGCACGAT-3' | Forward  Reverse |
| Human *Cd36* | 5'-TGCAAAGAAGGGAGACCTGTG-3'  5'-GTTGACCTGCAGCCGTTTTG-3' | Forward  Reverse |
| Human *Acaca* | 5'-TCACACCTGAAGACCTTAAAGCC-3'  5'-AGCCCACACTGCTTGTACTG-3' | Forward  Reverse |
| Human *Fasn* | 5'-ACAGCGGGGAATGGGTACT-3'  5'-GACTGGTACAACGAGCGGAT-3' | Forward  Reverse |
| Human *Scd1* | 5'-TCATAATTCCCGACGTGGCT-3'  5'-CCCAGAAATACCAGGGCACA-3' | Forward  Reverse |
| Human *Pparg* | 5'-TACTGTCGGTTTCAGAAATGCC-3'  5'-GTCAGCGGACTCTGGATTCAG-3' | Forward  Reverse |
| Human *Srebf1* | 5'-CCACTGGTCGTAGATGCG-3'  5'-GGGCTGCTCTGGAAAGG-3' | Forward  Reverse |
| Human *Ppara* | 5'-AGAGCCCCATCTGTCCTCTC-3'  5'-ACTGGTAGTCTGCAAAACCAAA-3' | Forward  Reverse |
| Human *Acox1* | 5'-GGAACTCACCTTCGAGGCTTG-3'  5'-TTCCCCTTAGTGATGAGCTGG-3' | Forward  Reverse |
| Human *Tnf* | 5'-TCAACCTCCTCTCTGCCATC-3'  5'-CCAAAGTAGACCTGCCCAGA-3' | Forward  Reverse |
| Human *Il6* | 5'-GAGTAGTGAGGAACAAGCCAGA-3'  5'-AAGCTGCGCAGAATGAGATGA-3' | Forward  Reverse |
| Human *Il8* | 5'-CTGGCCGTGGCTCTCTTG-3'  5'-GGGTGGAAAGGTTTGGAGTATG-3' | Forward  Reverse |
| Human *Il1b* | 5'-ACAGATGAAGTGCTCCTTCCA-3'  5'-GTCGGAGATTCGTAGCTGGAT-3' | Forward  Reverse |
| Human *Ccl5* | 5'-TCATTGCTACTGCCCTCTGC-3'  5'-TACTCCTTGATGTGGGCACG-3' | Forward  Reverse |
| Human *Cxcl10* | 5'-GTGGCATTCAAGGAGTACCTC-3'  5'-TGATGGCCTTCGATTCTGGATT-3' | Forward  Reverse |
| Human *Gper1* | 5'-GGGCCACGTCATGTCTCTAA-3'  5'-CTGGTCGACGGTGTCAGAAA-3' | Forward  Reverse |

**Figure S1**

**
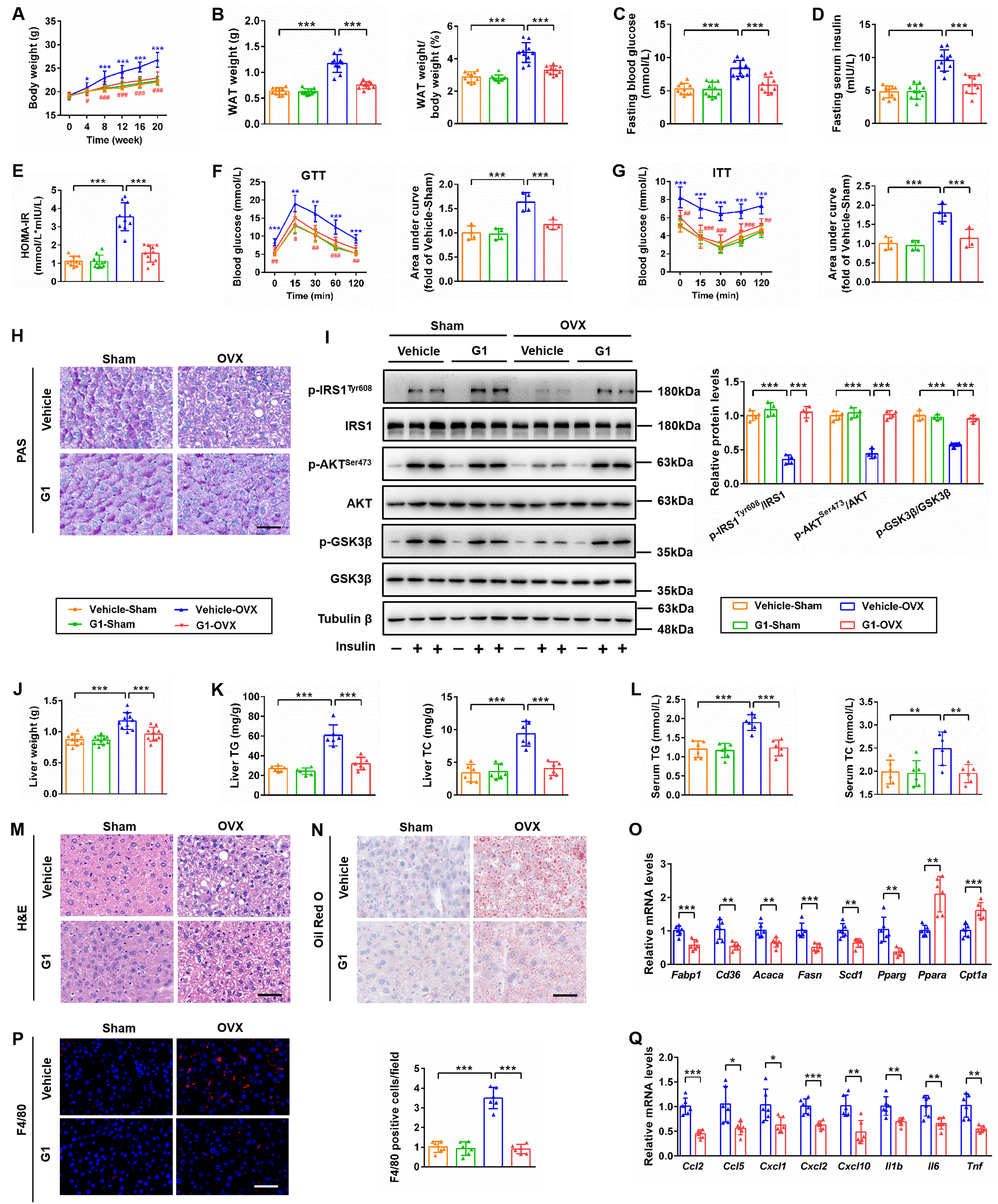
**

**Figure S1. Activation of GPER1 alleviates insulin resistance, hepatic lipid accumulation, and inflammation in OVX female mice**

(A) Body weight of sham or ovariectomized (OVX) female mice were treated with vehicle or GPER1-specific agonist G1 (5 mg/kg/2 days), accompanied by normal chow (NC) diet consumption from 0 to 20-week period (n = 10 mice/group).

(B) White adipose tissue (WAT) weight and ratios of WAT weight to body weight of the female mice in the indicated group (n = 10 mice/group).

(C-E) Fasting blood glucose levels (C), fasting blood insulin levels (D), and HOMA-IR values (E) of the female mice in the indicated group (n = 10 mice/group).

(F, G) Blood glucose levels after treatment with vehicle or G1 for 20 continuous weeks in sham or OVX female mice during intraperitoneal GTT (F) and intraperitoneal ITT (G). The corresponding areas under the curve are indicated on the right (n = 4 mice/group).

(H) Representative images of periodic acid-Schiff (PAS) staining on the liver sections of the female mice in the indicated group (n = 6 mice/group). Scale bar, 50 µm.

(I) Immunoblotting analyses of total and phosphorylated IRS1 (Tyr608), AKT (Ser473), and GSK3β protein levels in response to an intraperitoneal injection of saline or insulin (1.0 IU/kg for 15 min) on the liver tissues of the female mice in the indicated group (n = 4 mice/group). Tubulin β was served as the loading control. The immunoblot was quantified on the right.

(J) Liver weight of the female mice in the indicated group (n = 10 mice/group).

(K) Hepatic triglyceride (TG) and total cholesterol (TC) contents of the female mice in the indicated group (n = 6 mice/group).

(L) Serum TG and TC contents of the female mice in the indicated group (n = 6 mice/group).

(M, N) Representative images of hematoxylin-eosin (H&E) (M) and Oil Red O (N) staining on the liver sections of the female mice in the indicated group (n = 6 mice/group). Scale bar, 50 µm.

(O) Relative mRNA levels of factors related to fatty acid metabolism in the livers of the female mice in the indicated group (n = 6 mice/group).

(P) Representative images of F4/80 (red) immunofluorescence staining on the liver sections of the female mice in the indicated group, in which nuclei were stained with DAPI (blue). Scale bar, 50 µm. F4/80 positive cells in each field were quantified on the right (n = 6 mice/group).

(Q) Relative mRNA levels of pro-inflammatory factors in the livers of the female mice in the indicated group (n = 6 mice/group).

In all statistical plots, data are expressed as the mean ± SD. For statistical analysis, a two-tailed Student’s t-test was used for (O) and (Q), and one-way ANOVA with Bonferroni analysis was used for (A)-(G), (I)-(L), and (P). The mRNA expression of target genes was normalized to that of Actb. For (A), (F) (left), and (G) (left), *p < 0.05, **p < 0.01, ***p < 0.001, Vehicle-OVX group versus Vehicle-Sham group; ^#^p < 0.05, ^##^p < 0.01, ^###^p < 0.001, G1-OVX group versus Vehicle-OVX group. For (B)-(E), (F) (right), (G) (right), (I)-(L), and (O)-(Q), *p < 0.05, **p < 0.01, ***p < 0.001, comparison between the indicated groups.

**Figure S2**

**
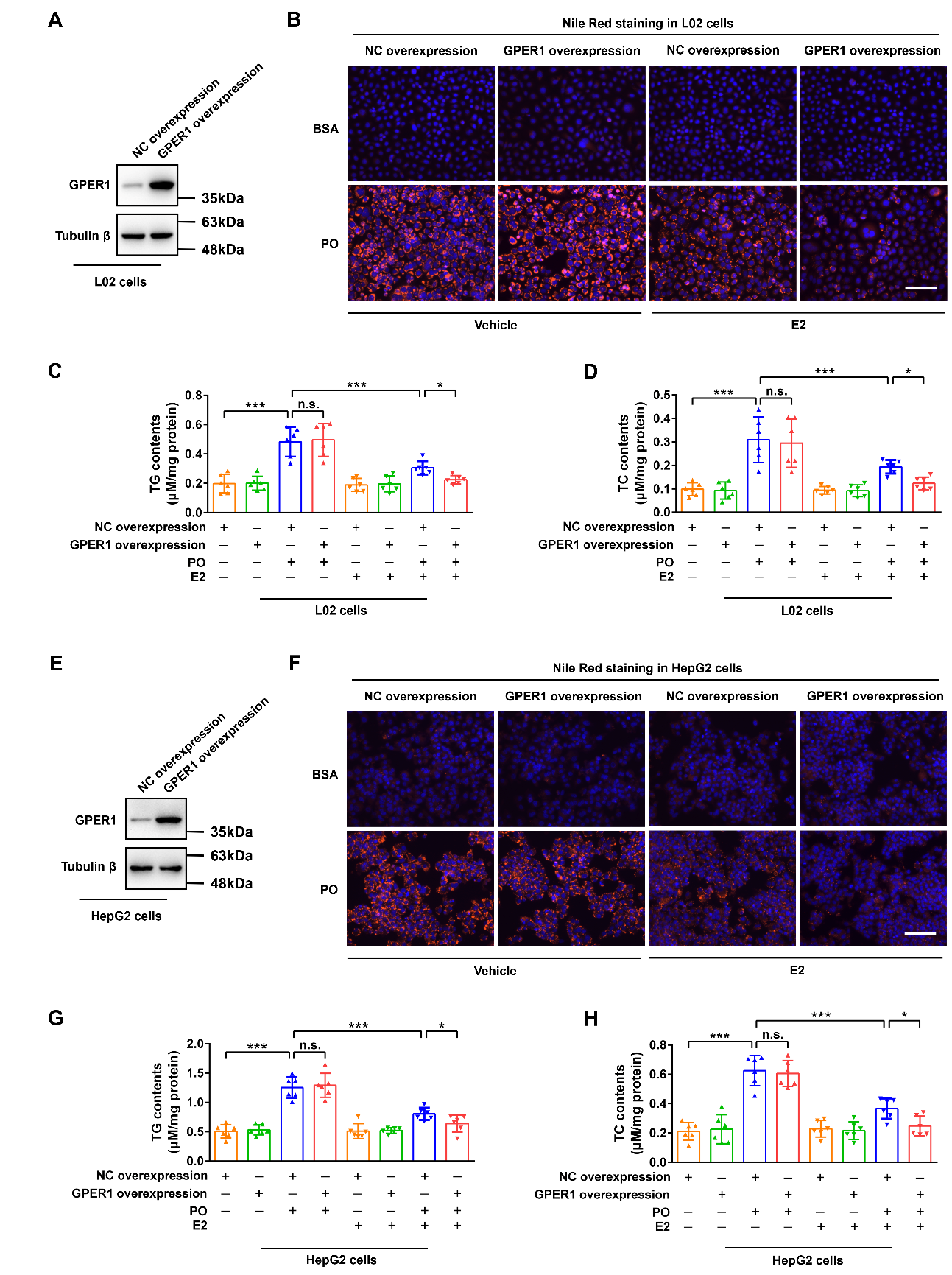
**

**Figure S2. Ligands are necessary for the overexpression of GPER1 to reduce lipid accumulation in hepatocytes**

(A) Immunoblotting analysis of GPER1 protein level in L02 cells transfected with control vector (NC overexpression) or GPER1 gene overexpression vector (GPER1 overexpression) (n = 3 independent experiments).

(B) Representative images of Nile Red stained L02 cells transfected with control vector (NC overexpression) or GPER1 gene overexpression vector (GPER1 overexpression) followed by vehicle or 17β-estradiol (E2; 10 nM) stimulation, and then treated with BSA or PO (palmitic acid and oil acid mixture) stimulation for 12 h (n = 3 independent experiments). Scale bar, 100 μm.

(C) Triglyceride (TG) contents in L02 cells from the indicated group (n = 6).

(D) Total cholesterol (TC) contents in L02 cells from the indicated group (n = 6).

(E) Immunoblotting analysis of GPER1 protein level in HepG2 cells transfected with control vector (NC overexpression) or GPER1 gene overexpression vector (GPER1 overexpression) (n = 3 independent experiments).

(F) Representative images of Nile Red stained HepG2 cells transfected with control vector (NC overexpression) or GPER1 gene overexpression vector (GPER1 overexpression) followed by vehicle or 17β-estradiol (E2; 10 nM) stimulation and then treated with BSA or PO for 12 h (n = 3 independent experiments). Scale bar, 100 μm.

(G) TG contents in HepG2 cells from the indicated group (n = 6).

(H) TC contents in HepG2 cells from the indicated group (n = 6).

In all statistical plots, data are expressed as the mean ± SD and analyzed by one-way ANOVA with Bonferroni analysis. *p < 0.05 and ***p < 0.001, comparison between the indicated groups; n.s., no significance, p ≥ 0.05, comparison between the indicated groups.

**Figure S3**

**
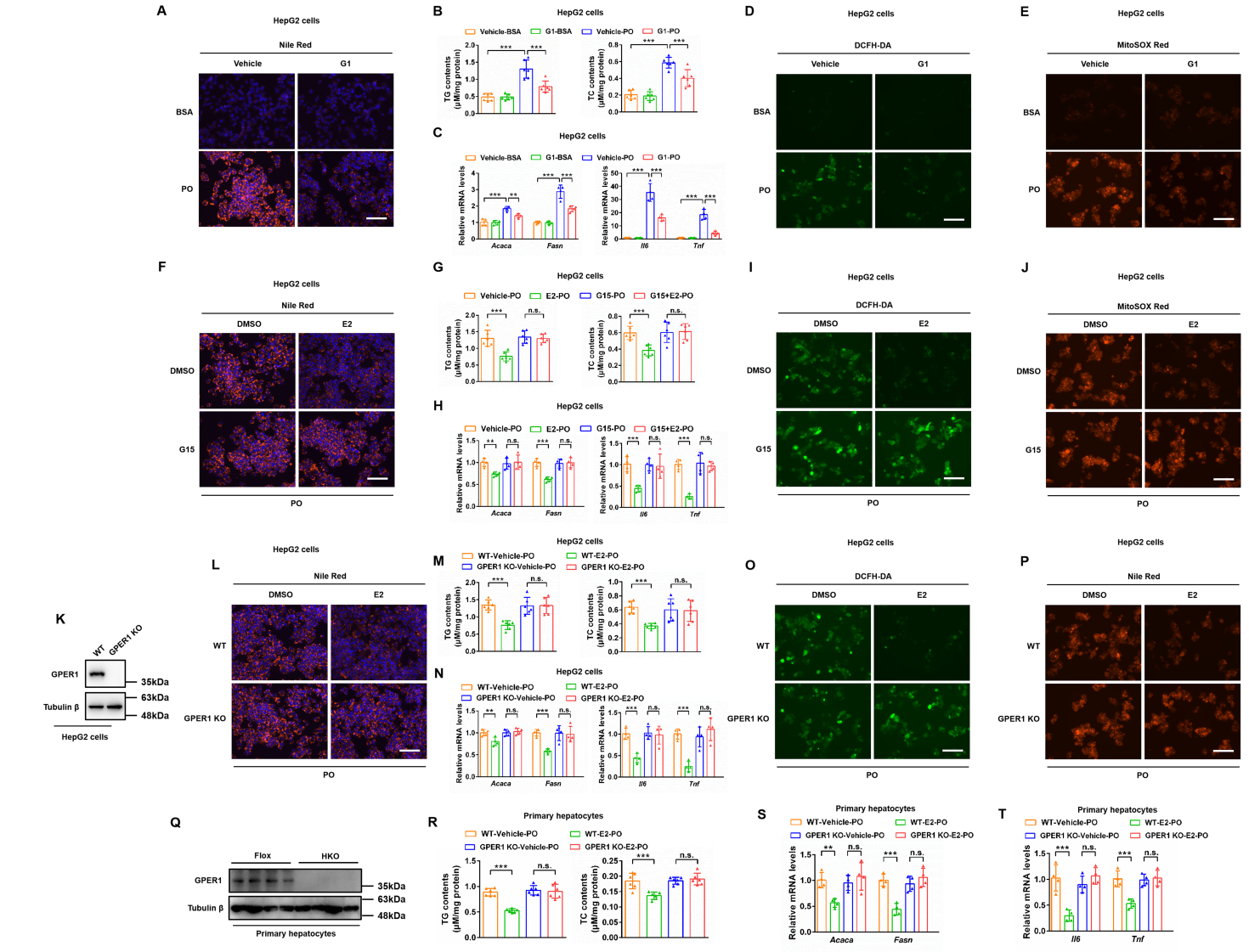
**

**Figure S3. GPER1 plays a crucial role in the protective roles of estradiol on lipid accumulation, oxidative stress, and inflammation in HepG2 cells and primary mouse hepatocytes**

(A) Representative images of Nile Red staining in HepG2 cells treated with vehicle or GPER1-specific agonist G1 (100 nM) followed by BSA or PO (palmitic acid and oil acid mixture) stimulation for 12 h (n = 3 independent experiments). Scale bar, 100 μm.

(B) Triglyceride (TG) and total cholesterol (TC) contents in HepG2 cells from the indicated group (n = 6).

(C) Relative mRNA levels of factors related to fatty acid metabolism (left) and inflammatory response (right) in HepG2 cells from the indicated group (n = 4).

(D) Representative images of DCFH-DA probe stained HepG2 cells in the indicated group (n = 3 independent experiments). Scale bar, 100 μm.

(E) Representative images of MitoSOX Red probe stained HepG2 cells in the indicated group (n = 3 independent experiments). Scale bar, 100 μm.

(F) Representative images of Nile Red staining in HepG2 cells challenged by PO and treated with vehicle, 17β-estradiol (E2; 10 nM), G15 (10 μM) or E2 in combination with G15 (n = 3 independent experiments). Scale bar, 100 μm.

(G) TG and TC contents in HepG2 cells from the indicated group (n = 6).

(H) Relative mRNA levels of genes related to fatty acid metabolism (left) and pro-inflammation factors (right) in HepG2 cells from the indicated group (n = 4).

(I) Representative images of DCFH-DA probe stained HepG2 cells in the indicated group (n = 3 independent experiments). Scale bar, 100 μm.

(J) Representative images of MitoSOX Red probe stained HepG2 cells in the indicated group (n = 3 independent experiments). Scale bar, 100 μm.

(K) Immunoblotting analysis of GPER1 protein level in the wild type (WT) and GPER1 knockout (KO) HepG2 cells (n = 3 independent experiments).

(L) Representative images of Nile Red staining in the WT and GPER1 KO HepG2 cells challenged by PO and co-treated with vehicle or E2 (n = 3 independent experiments). Scale bar, 100 μm.

(M) TG and TC contents in HepG2 cells from the indicated group (n = 6).

(N) Relative mRNA levels of factors related to fatty acid metabolism (left) and inflammatory response (right) in HepG2 cells from the indicated group (n = 4).

(O) Representative images of DCFH-DA probe stained HepG2 cells in the indicated group (n = 3 independent experiments). Scale bar, 100 μm.

(P) Representative images of MitoSOX Red probe stained HepG2 cells in the indicated group (n = 3 independent experiments). Scale bar, 100 μm.

(Q) Immunoblotting analysis of GPER1 protein expression level in primary hepatocytes that isolates from the GPER1-Flox control and GPER1-HKO female mice (n = 4). Tubulin β was served as the loading control.

(R) TG and TC contents in primary hepatocytes that isolates from the GPER1-Flox control and GPER1-HKO female mice challenged by PO (palmitic acid and oil acid mixture) and co-treated with vehicle or E2 (10 nM) (n = 6).

(S, T) Relative mRNA levels of factors related to fatty acid metabolism (S) and inflammatory response (T) in primary hepatocytes from the indicated group (n = 4).

In all statistical plots, data are expressed as the mean ± SD and analyzed by one-way ANOVA with Bonferroni analysis. **p < 0.01 and ***p < 0.001, comparison between the indicated groups; n.s., no significance, p ≥ 0.05, comparison between the indicated groups. The mRNA expression of target genes was normalized to that of Actb.

**Figure S4**

**
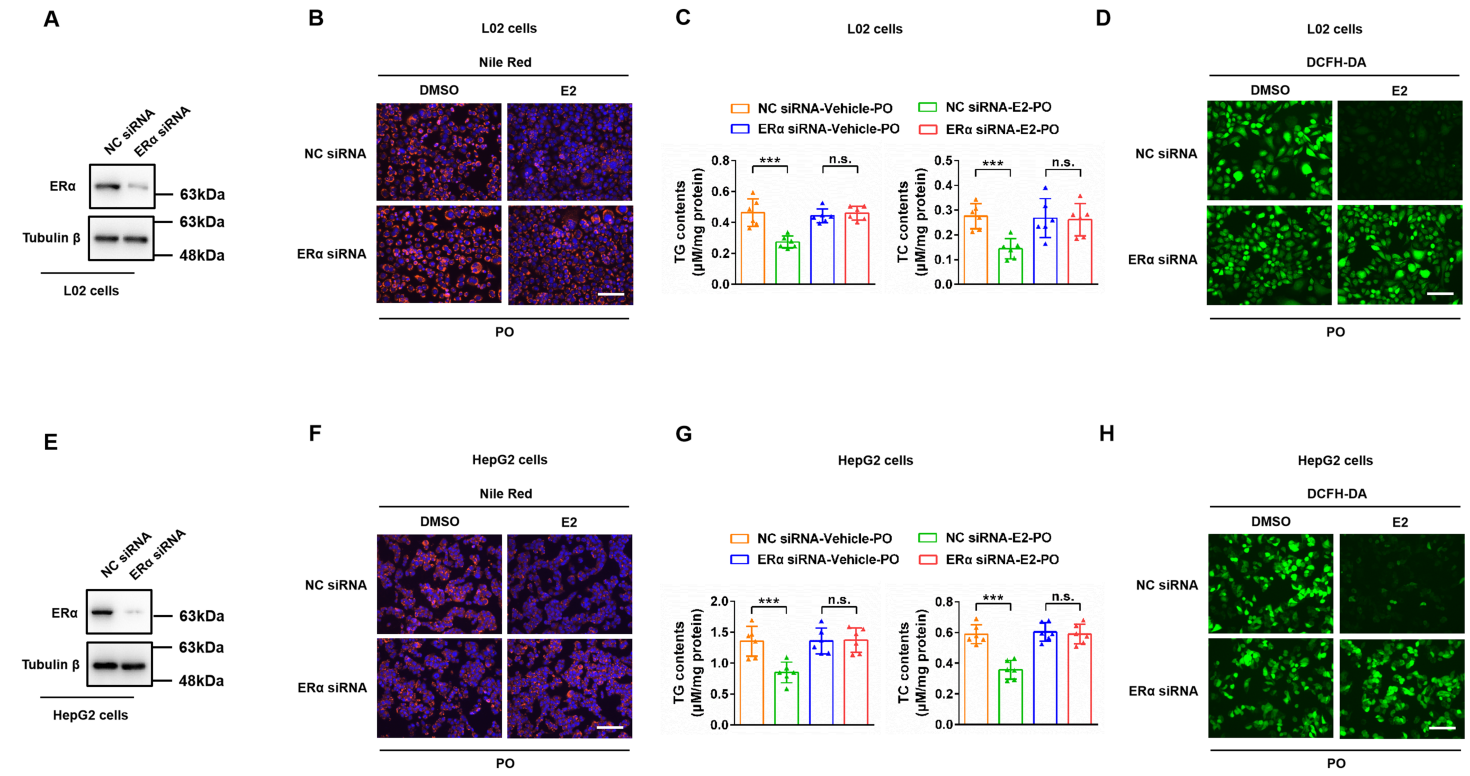
**

**Figure S4.** **ERα plays a crucial role in the protective roles of estradiol on lipid accumulation and oxidative stress in L02 cells and HepG2 cells**

(A) Immunoblotting analysis of ERα protein level in the L02 cells transfected with siRNA targeting normal control (NC) or ERα (n = 3 independent experiments).

(B) Representative images of Nile Red staining in L02 cells challenged by PO (palmitic acid and oil acid mixture) and co-treated with vehicle or E2 (n = 3 independent experiments). Scale bar, 100 μm.

(C) Triglyceride (TG) and total cholesterol (TC) contents in L02 cells from the indicated group (n = 6).

(D) Representative images of DCFH-DA probe stained L02 cells in the indicated group (n = 3 independent experiments). Scale bar, 100 μm.

(E) Immunoblotting analysis of ERα protein level in the HepG2 cells transfected with siRNA targeting normal control (NC) or ERα (n = 3 independent experiments).

(F) Representative images of Nile Red staining in HepG2 cells challenged by PO and co-treated with vehicle or E2 (n = 3 independent experiments). Scale bar, 100 μm.

(G) TG and TC contents in HepG2 cells from the indicated group (n = 6).

(H) Representative images of DCFH-DA probe stained HepG2 cells in the indicated group (n = 3 independent experiments). Scale bar, 100 μm.

In all statistical plots, data are expressed as the mean ± SD and analyzed by one-way ANOVA with Bonferroni analysis. ***p < 0.001, comparison between the indicated groups; n.s., no significance, p ≥ 0.05, comparison between the indicated groups.

**Figure S5**

**
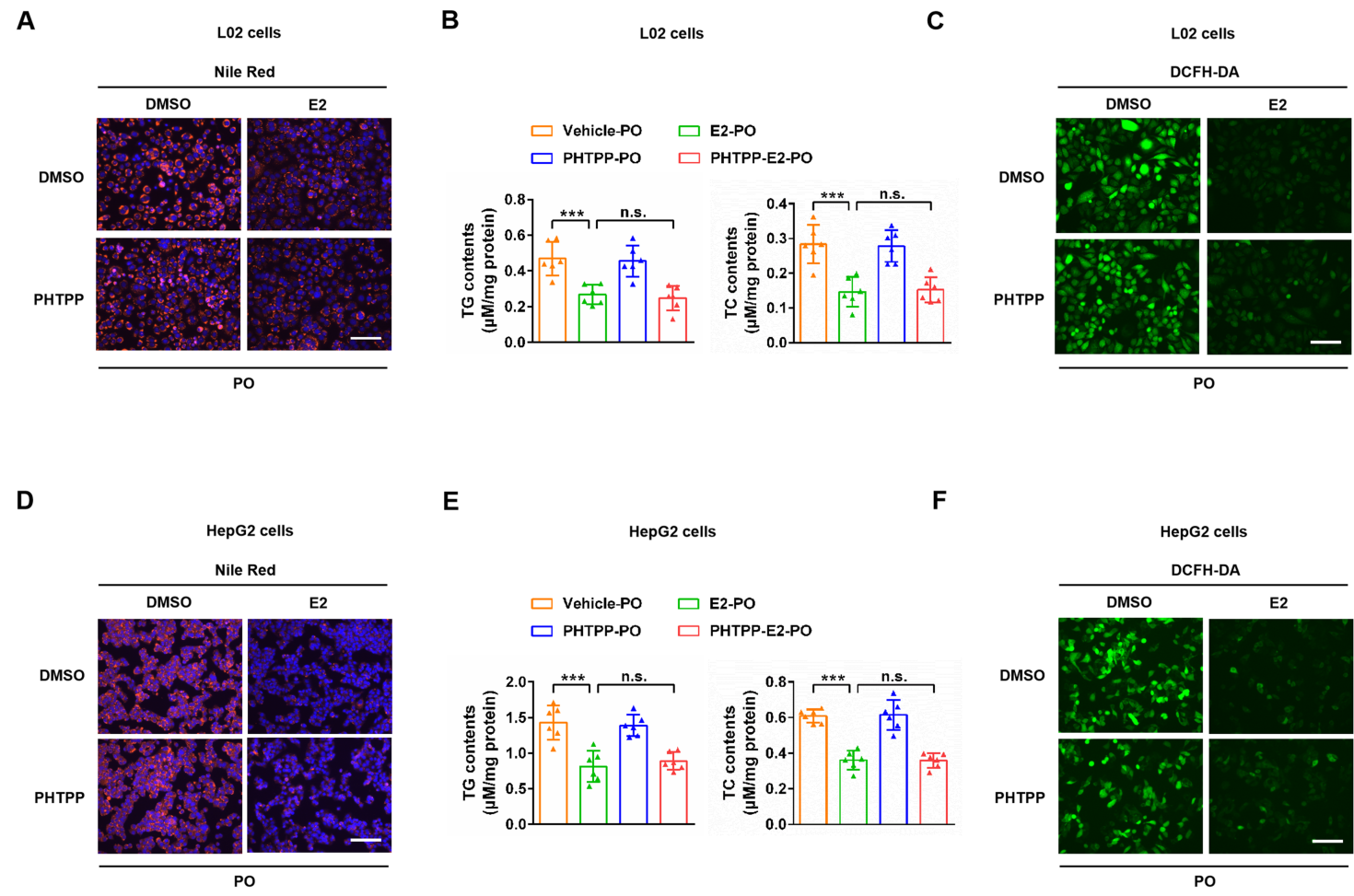
**

**Figure S5. Inhibition of ERβ did not affect the beneficial effects of E2 on PO-induced lipid accumulation and oxidative stress in hepatocytes**

(A) Representative images of Nile Red staining in L02 cells challenged by PO (palmitic acid and oil acid mixture) and treated with vehicle, 17β-estradiol (E2; 10 nM), PHTPP (10 μM) or E2 in combination with PHTPP (n = 3 independent experiments). Scale bar, 100 μm.

(B) Triglyceride (TG) and total cholesterol (TC) contents in L02 cells from the indicated group (n = 6).

(C) Representative images of DCFH-DA probe stained L02 cells in the indicated group (n = 3 independent experiments). Scale bar, 100 μm.

(D) Representative images of Nile Red staining in HepG2 cells challenged by PO and treated with vehicle, 17β-estradiol (E2; 10 nM), PHTPP (10 μM) or E2 in combination with PHTPP (n = 3 independent experiments). Scale bar, 100 μm.

(E) TG and TC contents in HepG2 cells from the indicated group (n = 6).

(F) Representative images of DCFH-DA probe stained HepG2 cells in the indicated group (n = 3 independent experiments). Scale bar, 100 μm.

In all statistical plots, data are expressed as the mean ± SD and analyzed by one-way ANOVA with Bonferroni analysis. ***p < 0.001, comparison between the indicated groups; n.s., no significance, p ≥ 0.05, comparison between the indicated groups.

**Figure S6**

**
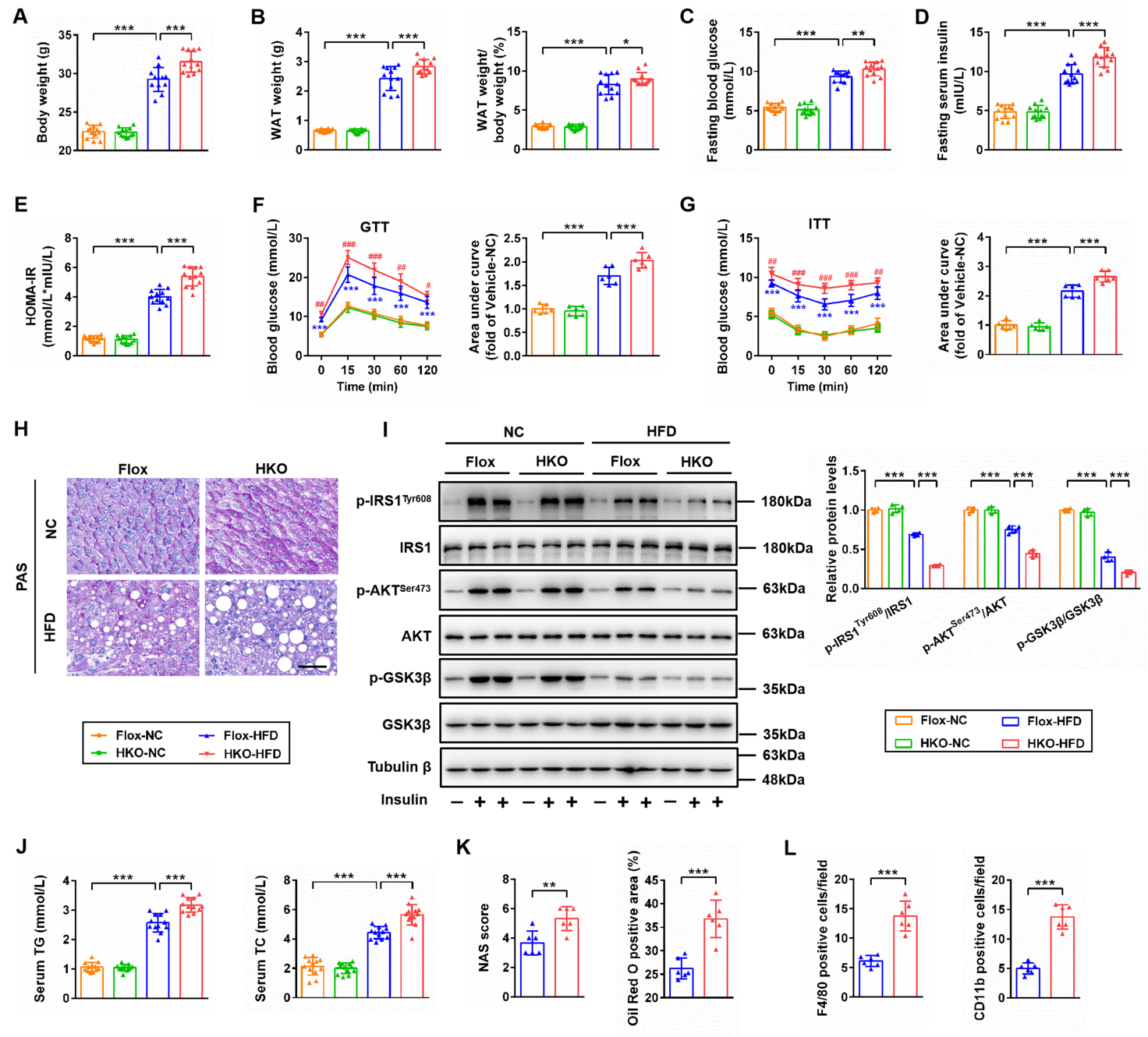
**

**Figure S6. Hepatocyte-specific GPER1 knockout aggravates HFD-induced insulin resistance in female mice, related to Figure 3**

(A) Body weight of GPER1-HKO female mice and their corresponding controls GPER1-Flox female mice after normal chow (NC) diet or high-fat diet (HFD) consumption for 24 weeks (n = 12 mice/group).

(B) White adipose tissue (WAT) weight and ratios of WAT weight to body weight of the female mice in the indicated group (n = 12 mice/group).

(C-E) Fasting blood glucose levels (C), fasting blood insulin levels (D), and HOMA-IR values (E) of the female mice in the indicated group (n = 12 mice/group).

(F, G) Blood glucose levels after NC or HFD feeding for 24 continuous weeks in GPER1-HKO female mice and their corresponding controls GPER1-Flox female mice during intraperitoneal GTT (F) and intraperitoneal ITT (G). The corresponding areas under the curve are indicated on the right (n = 6 mice/group).

(H) Representative images of periodic acid-Schiff (PAS) staining on the liver sections of the female mice in the indicated group (n = 6 mice/group). Scale bar, 50 µm.

(I) Immunoblotting analyses of total and phosphorylated IRS1 (Tyr608), AKT (Ser473), and GSK3β protein level in response to an intraperitoneal injection of saline or insulin (1.0 IU/kg for 15 min) in the liver tissues of the female mice in the indicated group (n = 4 mice/group). Tubulin β was served as the loading control. The immunoblot was quantified on the right.

(J) Serum triglyceride (TG) and total cholesterol (TC) contents of the female mice in the indicated group (n = 12 mice/group).

(K) Quantification of hematoxylin-eosin (H&E) (upper) and Oil Red O (lower) staining in Figure 3C

(L) Quantification of CD11b (upper) and F4/80 (lower) immunofluorescence staining in Figure 3F

In all statistical plots, data are expressed as the mean ± SD. For statistical analysis, a two-tailed Student’s t-test was used for (K) and (L), and one-way ANOVA with Bonferroni analysis was used for (A-(G), (I), and (J). For (F) (left) and (G) (left), ***p < 0.001, Flox-HFD group versus Flox-NC group; ^#^p < 0.05, ^##^p < 0.01, ^###^p < 0.001, HKO-HFD group versus Flox-HFD group. For (A)-(E), (F) (right), (G) (right), and (I)-(L), *p < 0.05, **p < 0.01, ***p < 0.001, comparison between the indicated groups.

**Figure S7**

**
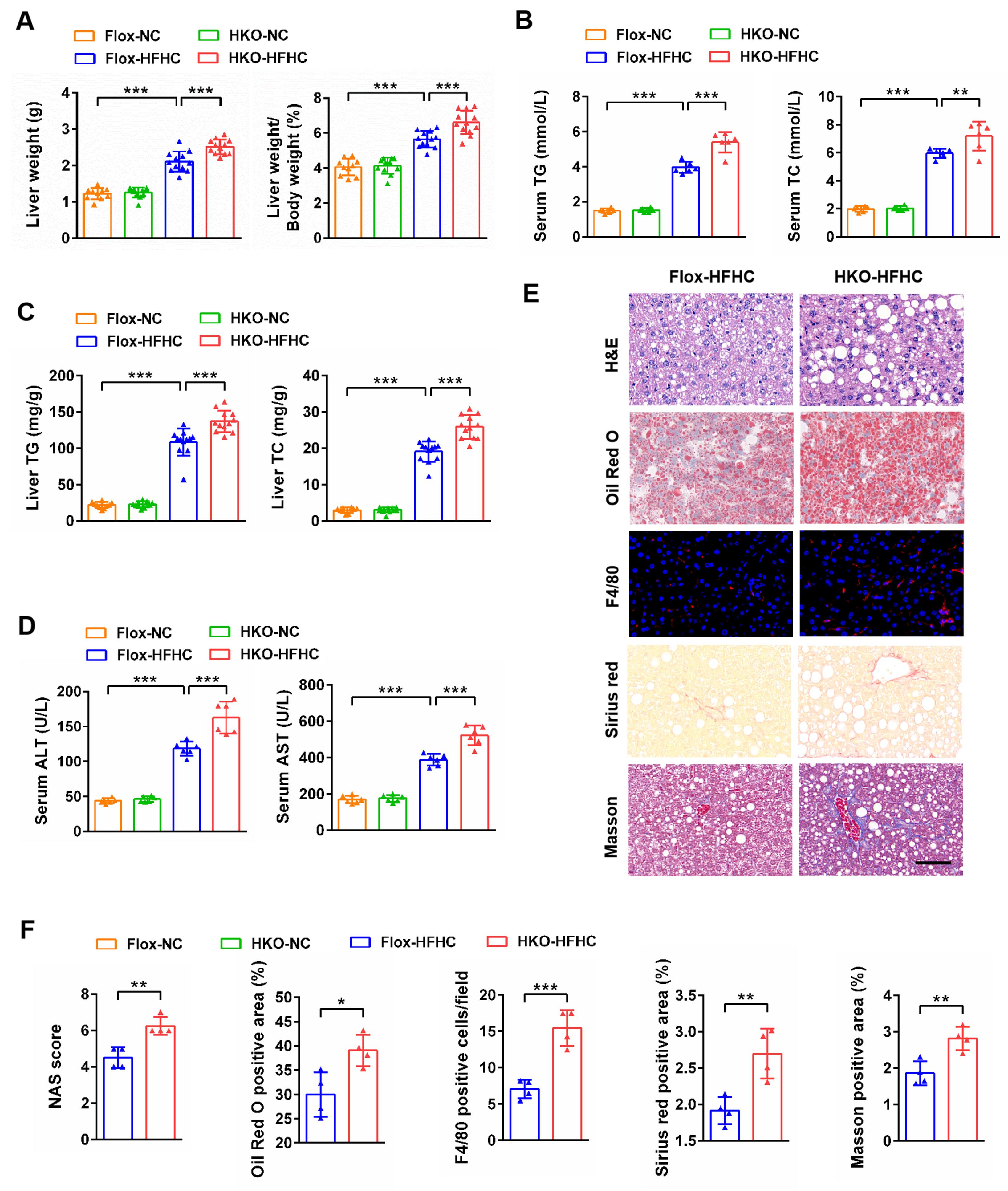
**

**Figure S7. Hepatocyte-specific GPER1 knockout exacerbates the hepatic steatosis, inflammation, and fibrosis induced by HFHC diet in male mice**

(A) Liver weight and ratios of liver weight to body weight of the GPER1-HKO male mice and their corresponding controls GPER1-Flox male mice after NC or high-fat and high-cholesterol (HFHC) diet consumption for 16 weeks (n = 10-12 mice/group).

(B) Serum triglyceride (TG) and total cholesterol (TC) contents of the male mice in the indicated group (n = 6 mice/group).

(C) Hepatic TG and TC contents of the male mice in the indicated group (n = 10-12 mice/group).

(D) Serum alanine aminotransferase (ALT) and aspartate aminotransferase (AST) activities of the male mice in the indicated group (n = 6 mice/group).

(E) Representative images of hematoxylin-eosin (H&E), Oil Red O, F4/80, Sirius red, and Masson staining on the liver sections of the male mice in the indicated group (n = 4 mice/group). Scale bar, 50 μm.

(F) Quantification of H&E, Oil Red O, F4/80, Sirius red, and Masson staining in Figure S7E (n = 4 mice/group).

In all statistical plots, data are expressed as the mean ± SD. For statistical analysis, a two-tailed Student’s t-test was used for (F), and one-way ANOVA with Bonferroni analysis was used for (A)-(D). *p < 0.05, **p < 0.01, ***p < 0.001, comparison between the indicated groups.

**Figure S8**

**
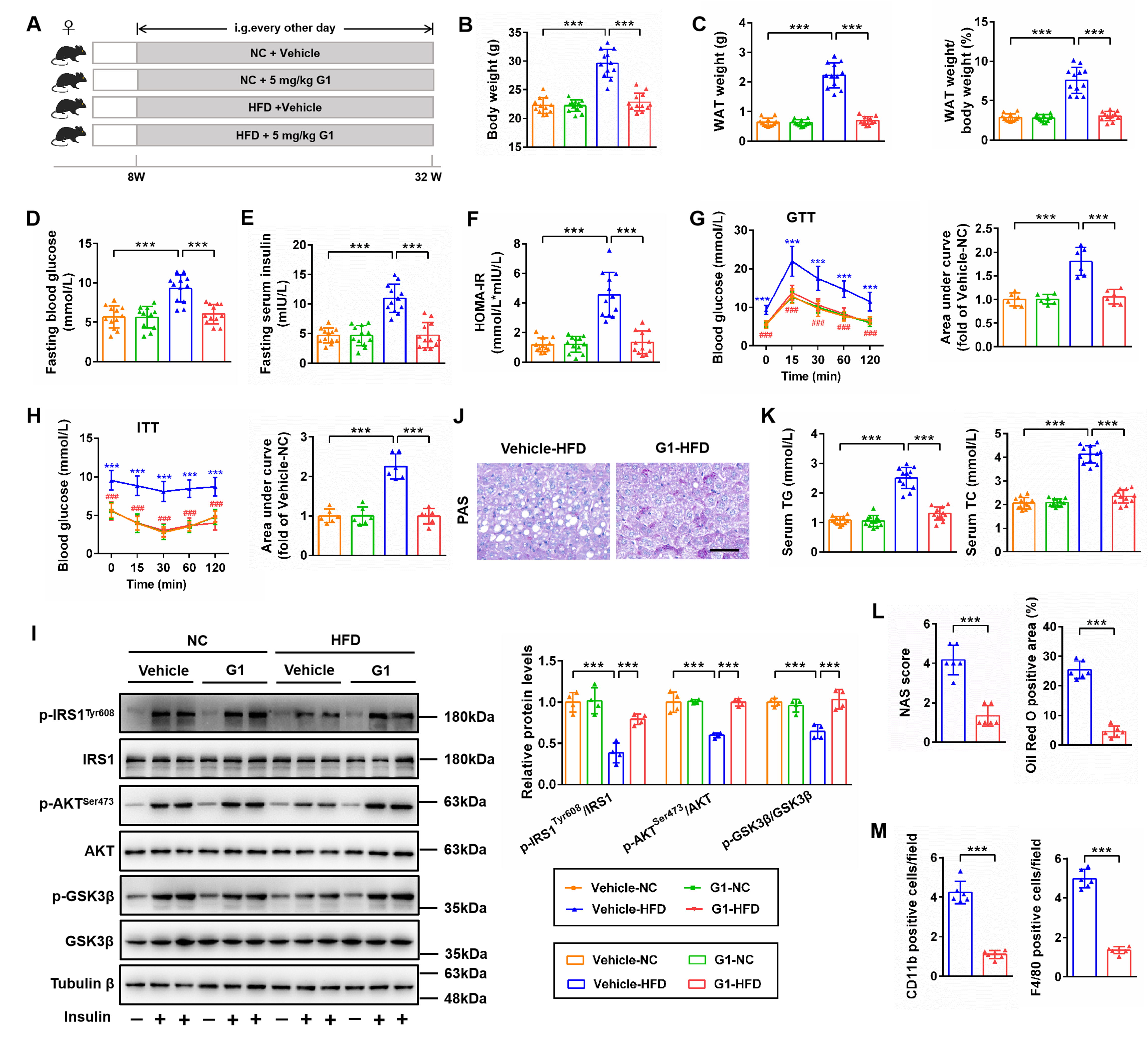
**

**Figure S8. GPER1-specific agonist G1 attenuates HFD-induced insulin resistance in female mice, related to Figure 4**

(A) Scheme for the experimental strategy on normal chow (NC) diet- or high-fat diet (HFD)-fed female mice treated with vehicle or GPER1-specific agonist G1 (5 mg/kg/2 days) for 24 weeks.

(B) Body weight of the female mice in the indicated group (n = 12 mice/group).

(C) White adipose tissue (WAT) weight and ratios of WAT weight to body weight of the female mice in the indicated group (n = 12 mice/group).

(D-F) Fasting blood glucose levels (D), fasting blood insulin levels (E), and HOMA-IR values (F) of the female mice in the indicated group (n = 12 mice/group).

(G, H) Blood glucose levels after treatment with vehicle or G1 for 24 continuous weeks in NC- or HFD-fed-diet female mice during intraperitoneal GTT (G) and intraperitoneal ITT (H). The corresponding areas under the curve are indicated on the right (n = 6 mice/group).

(I) Immunoblotting analyses of total and phosphorylated IRS1 (Tyr608), AKT (Ser473), and GSK3β protein level in response to an intraperitoneal injection of saline or insulin (1.0 IU/kg for 15 min) in the liver tissues of the female mice in the indicated group. Tubulin β was served as the loading control. The immunoblot was quantified on the right (n = 4 mice/group).

(J) Representative images of periodic acid-Schiff (PAS) staining on the liver sections of the female mice in the indicated group (n = 6 mice/group). Scale bar, 50 µm.

(K) Serum triglyceride (TG) and total cholesterol (TC) contents of the female mice in the indicated group (n = 12 mice/group).

(L) Quantification of hematoxylin-eosin (H&E) (upper) and Oil Red O (lower) staining in Figure 4C.

(M) Quantification of CD11b (upper) and F4/80 (lower) immunofluorescence staining in Figure 4F.

In all statistical plots, data are expressed as the mean ± SD. For statistical analysis, a two-tailed Student’s t-test was used for (L) and (M), and one-way ANOVA with Bonferroni analysis was used for (B)-(I) and (K). For (G) (left) and (H) (left), ***p < 0.001, Vehicle-HFD group versus Vehicle-NC group; ^###^p < 0.001, G1-HFD group versus Vehicle-HFD group. For (B)-(F), (G) (right), (H) (right), (I), and (K)-(M), ***p < 0.001, comparison between the indicated groups.

**Figure S9**

**
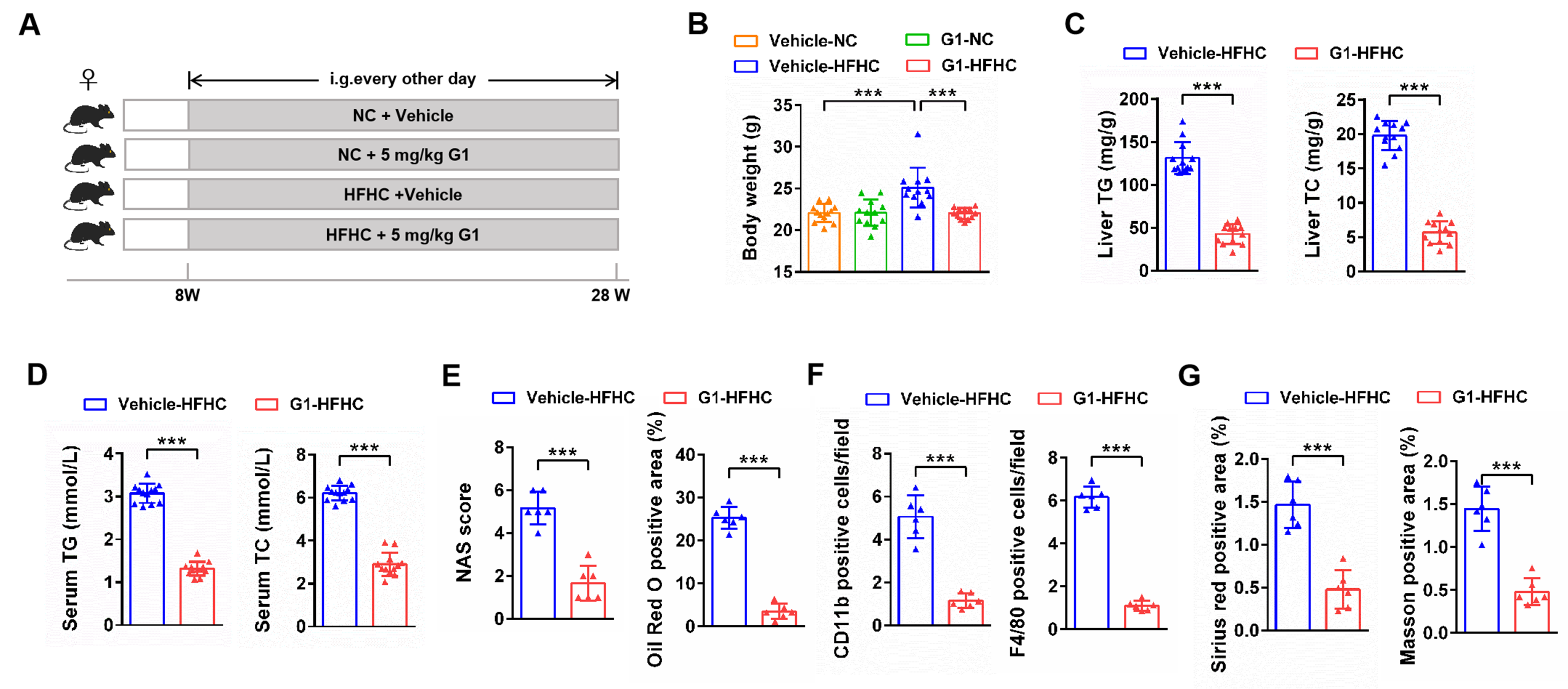
**

**Figure S9. GPER1-specific agonist G1 ameliorates HFHC-induced metabolic dysfunction in female mice, related to Figure 4**

(A) Scheme for the experimental strategy on normal chow (NC) diet- or high-fat and high-cholesterol (HFHC)-diet-fed female mice treated with vehicle or GPER1-specific agonist G1 (5 mg/kg/2 days) for 20 weeks.

(B) Body weight of the female mice in the indicated group (n = 12 mice/group).

(C) Hepatic triglyceride (TG) and total cholesterol (TC) contents of the female mice in the indicated group (n = 12 mice/group).

(D) Serum TG and TC contents of the female mice in the indicated group (n = 12 mice/group).

(E) Quantification of hematoxylin-eosin (H&E) (upper) and Oil Red O (lower) staining in Figure 4J (n = 6 mice/group).

(F) Quantification of CD11b (upper) and F4/80 (lower) immunofluorescence staining in Figure 4M (n = 6 mice/group).

(G) Quantification of Sirius red (upper) and Masson (lower) staining in Figure 4O (n = 6 mice/group).

In all statistical plots, data are expressed as the mean ± SD. For statistical analysis, a two-tailed Student’s t-test was used for (C)-(G), and one-way ANOVA with Bonferroni analysis was used for (B). ***p < 0.001, comparison between the indicated groups.

**Figure S10**

**
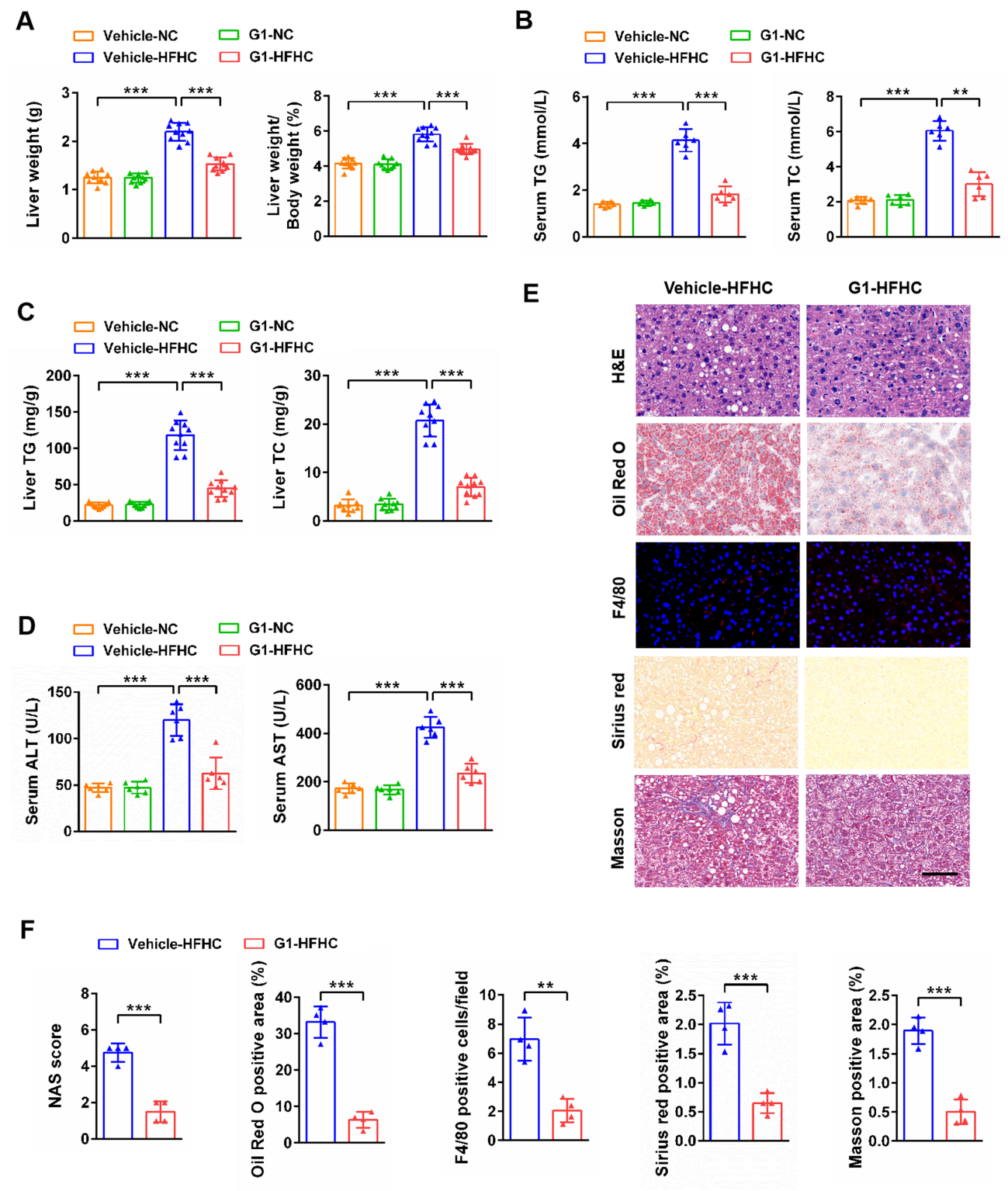
**

**Figure S10. Activation of GPER1 by G1 mitigates HFHC-induced hepatic steatosis, inflammation, and fibrosis in male mice**

(A) Liver weight and ratios of liver weight to body weight of GPER1-HKO male mice and their corresponding controls GPER1-Flox male mice after NC or high-fat and high-cholesterol (HFHC) diet consumption for 16 weeks (n = 9-10 mice/group).

(B) Serum triglyceride (TG) and total cholesterol (TC) contents of the male mice in the indicated group (n = 6 mice/group).

(C) Hepatic TG and TC contents of the male mice in the indicated group (n = 9-10 mice/group).

(D) Serum alanine aminotransferase (ALT) and aspartate aminotransferase (AST) activities of the male mice in the indicated group (n = 6 mice/group).

(E) Representative images of hematoxylin-eosin (H&E), Oil Red O, F4/80, Sirius red, and Masson staining on the liver sections of the male mice in the indicated group (n = 4 mice/group). Scale bar, 50 μm.

(F) Quantification of H&E, Oil Red O, F4/80, Sirius red, and Masson staining in Figure S10E (n = 4 mice/group).

In all statistical plots, data are expressed as the mean ± SD. For statistical analysis, a two-tailed Student’s t-test was used for (F), and one-way ANOVA with Bonferroni analysis was used for (A)-(D). **p < 0.01 and ***p < 0.001, comparison between the indicated groups

**Figure S11**

**
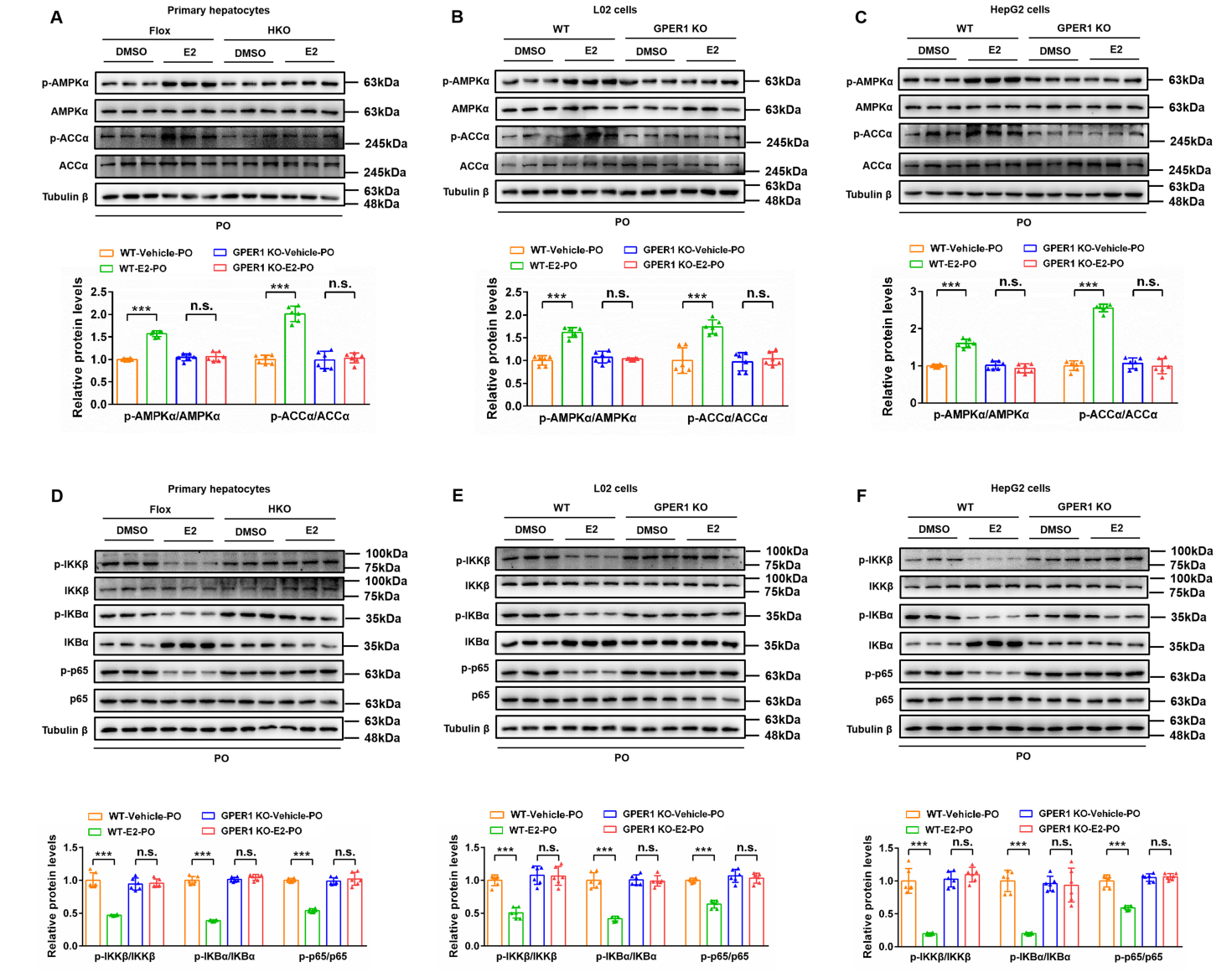
**

**Figure S11. GPER1 regulates AMPK and its downstream pathways during lipid accumulation, oxidative stress, and inflammation in PO-induced hepatocytes.**

(A) Immunoblotting analyses of total and phosphorylated AMPKα and ACCα protein levels in primary hepatocytes that isolate from GPER1-Flox control or GPER1-HKO female mice challenged by PO (palmitic acid and oil acid mixture) and co-treated with vehicle or 17β-estradiol (E2; 10 nM) for 12 h.

(B) Immunoblotting analyses of total and phosphorylated AMPKα and ACCα protein levels in wild type (WT) and GPER1 knockout (KO) L02 cells challenged by PO and co-treated with vehicle or E2 for 12 h.

(C) Immunoblotting analyses of total and phosphorylated AMPKα and ACCα protein levels in WT and GPER1 KO HepG2 cells challenged by PO and co-treated with vehicle or E2 for 12 h.

(D) Immunoblotting analyses of total and phosphorylated IKKβ, IKBα, and p65 protein levels in primary hepatocytes that isolate from GPER1-Flox control or GPER1-HKO female mice challenged by PO and co-treated with vehicle or E2 for 12 h.

(E) Immunoblotting analyses of total and phosphorylated IKKβ, IKBα, and p65 protein levels in WT and GPER1 KO L02 cells challenged by PO and co-treated with vehicle or E2 for 12 h.

(F) Immunoblotting analyses of total and phosphorylated IKKβ, IKBα, and p65 in WT and GPER1 KO HepG2 cells challenged by PO and treated with vehicle or E2 for 12 h.

In all statistical plots, data are expressed as the mean ± SD and analyzed by one-way ANOVA with Bonferroni analysis. ***p < 0.001, comparison between the indicated groups; n.s., no significance, p ≥ 0.05, comparison between the indicated groups. For immunoblotting, tubulin β was served as the loading control.

**Figure S12**

**
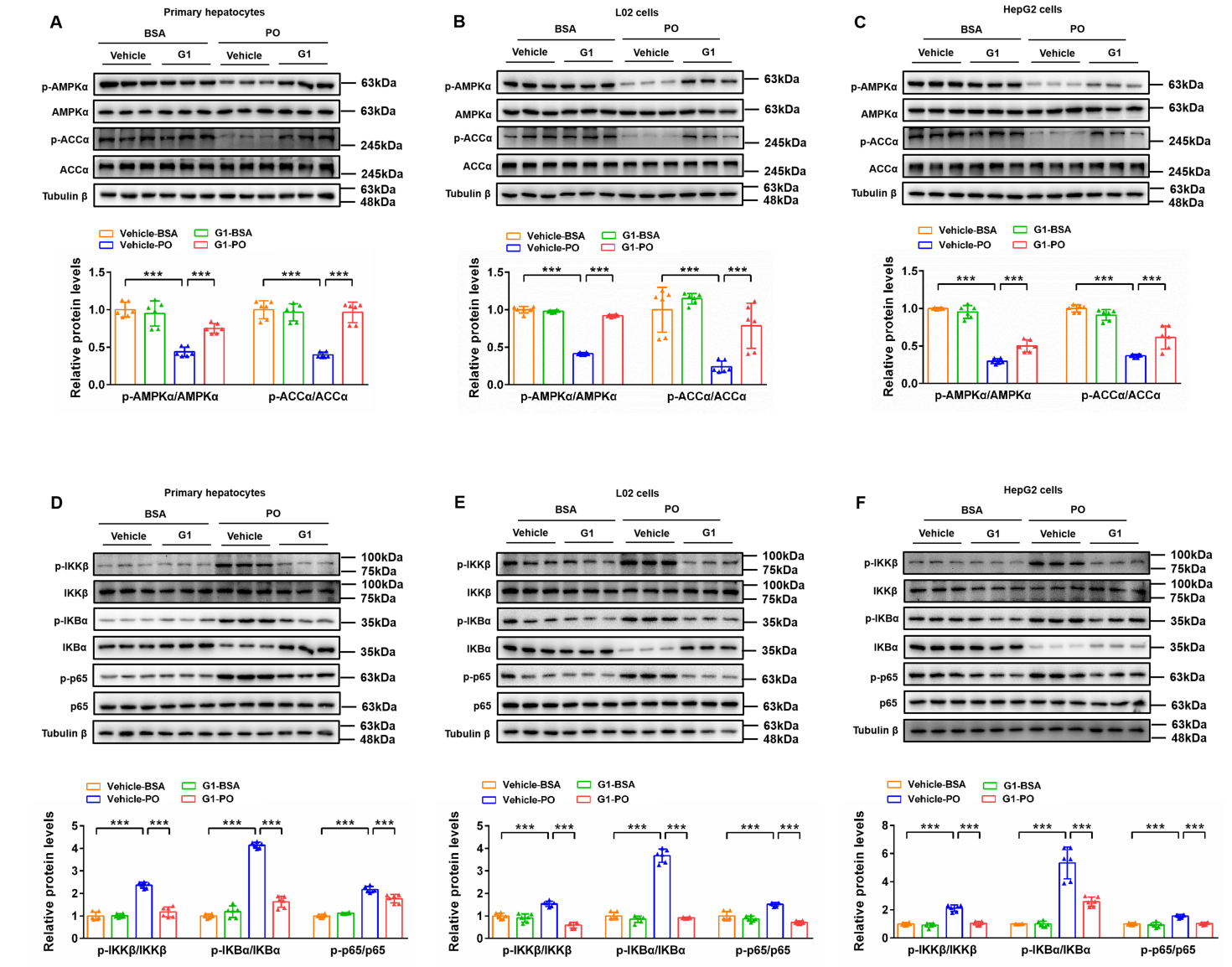
**

**Figure S12. AMPK and its downstream pathways are involved in GPER1-regulated development of lipid accumulation, oxidative stress, and inflammation in hepatocytes**

(A) Immunoblotting analyses of total and phosphorylated AMPKα and ACCα protein levels in primary hepatocytes that isolate from female mice challenged by BSA or PO (palmitic acid and oil acid mixture) and co-treated with vehicle or 100 nM GPER1-specific agonist G1 for 12 h.

(B) Immunoblotting analyses of total and phosphorylated AMPKα and ACCα protein levels in L02 cells challenged by BSA or PO and treated with vehicle or 100 nM G1 for 12 h.

(C) Immunoblotting analyses of total and phosphorylated AMPKα and ACCα protein levels in HepG2 cells challenged by BSA or PO and treated with vehicle or 100 nM G1 for 12 h.

(D) Immunoblotting analyses of total and phosphorylated IKKβ, IKBα, and p65 protein levels in primary hepatocytes that isolate from female mice challenged by BSA or PO and treated with vehicle or 100 nM G1 for 12 h.

(E) Immunoblotting analyses of total and phosphorylated IKKβ, IKBα, and p65 protein levels in L02 cells challenged by BSA or PO and treated with vehicle or 100 nM G1 for 12 h.

(F) Immunoblotting analyses of total and phosphorylated IKKβ, IKBα, and p65 protein levels in HepG2 cells challenged by BSA or PO and treated with vehicle or 100 nM G1 for 12 h.

In all statistical plots, data are expressed as the mean ± SD and analyzed by one-way ANOVA with Bonferroni analysis. ***p < 0.001, comparison between the indicated groups; n.s., no significance, p ≥ 0.05, comparison between the indicated groups. For immunoblotting, tubulin β was served as the loading control.

**Figure S13**

**
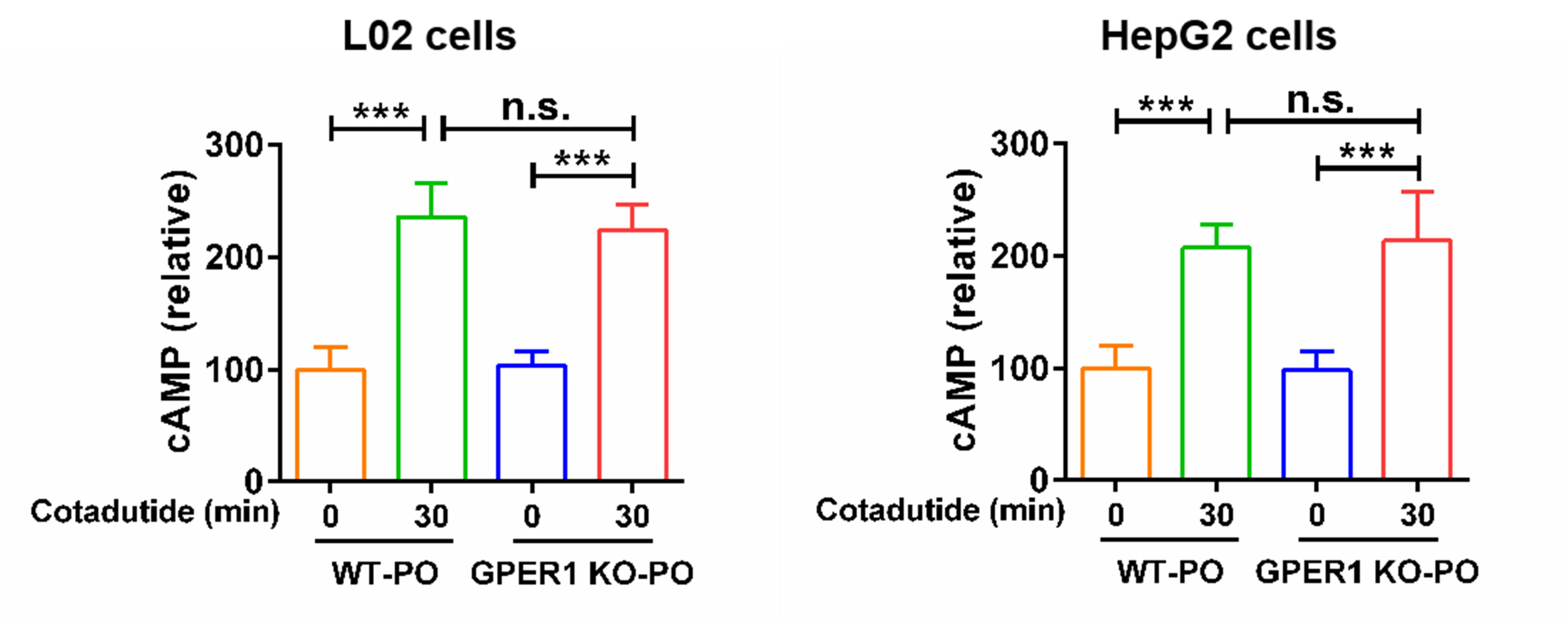
**

**Figure S13. GPER1 deletion did not affect cotadutide-stimulated cAMP production in PO-induced hepatocytes**

(A) Cyclic AMP levels in wild type (WT) or GPER1 knockout (KO) L02 cells were challenged by PO (palmitic acid and oil acid mixture) and co-treated with vehicle or cotadutide (GLP-1/GCGR agonist) for 30 min (n = 4).

(A) Cyclic AMP levels in WT or GPER1 KO HepG2 cells were challenged by PO and co-treated with vehicle or cotadutide for 30 min (n = 4).

In all statistical plots, data are expressed as the mean ± SD and analyzed by one-way ANOVA with Bonferroni analysis. ***p < 0.001, comparison between the indicated groups; n.s., no significance, p ≥ 0.05, comparison between the indicated groups.

**Figure S14**

**
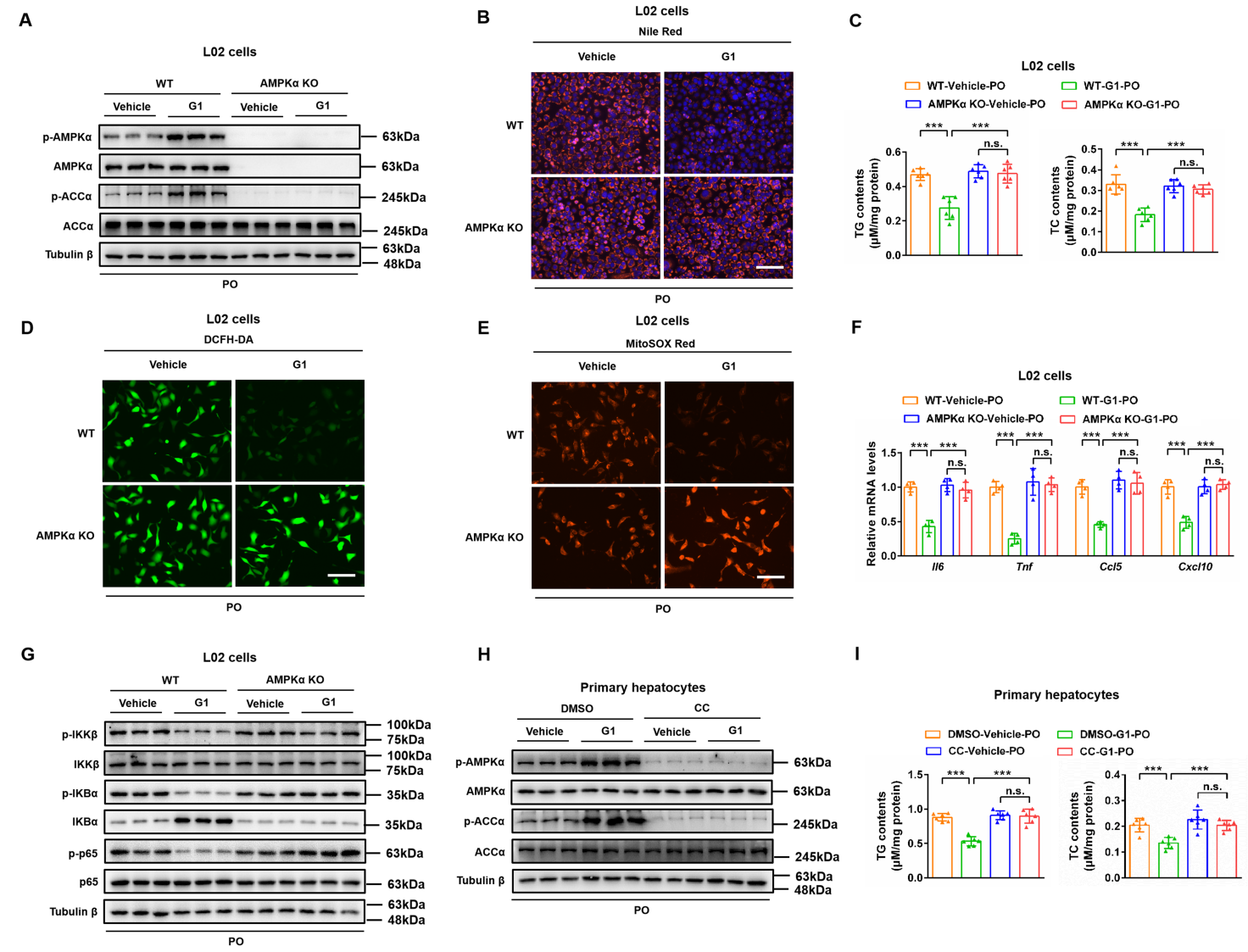
**

**Figure S14. AMPK activation is required in the beneficial effects of G1 on PO-induced lipid accumulation, oxidative stress, and inflammation in hepatocytes**

(A) Immunoblotting analyses of total and phosphorylated AMPKα and ACCα protein levels in wild type (WT) and PRKAA1/2 (encoding AMPKα1/α2) double-knockout (AMPKα KO) L02 cells treated with vehicle or GPER1-specific agonist G1 under PO (palmitic acid and oil acid mixture) stimulation (n = 6). Tubulin β was served as the loading control.

(B) Representative images of Nile Red stained L02 cells in the indicated group (n = 3 independent experiments). Scale bar, 100 μm.

(C) Triglyceride (TG) and total cholesterol (TC) contents of L02 cells in the indicated group (n = 6).

(D) Representative images of DCFH-DA probe stained L02 cells in the indicated group (n = 3 independent experiments). Scale bar, 100 μm.

(E) Representative images of MitoSOX Red probe stained L02 cells in the indicated group (n = 3 independent experiments). Scale bar, 100 μm.

(F) Relative mRNA levels of pro-inflammatory mediators of L02 cells in the indicated group (n = 4).

(G) Immunoblotting analyses of total and phosphorylated IKKβ, IKBα, and p65 protein levels of L02 cells in the indicated group (n = 6). Tubulin β was served as the loading control.

(H) Immunoblotting analyses of total and phosphorylated AMPKα and ACCα protein levels in PO-simulated primary hepatocytes that were isolated from the wide type (WT) female mice treated with vehicle or G1 in the absence or presence of compound C (CC) (n = 6). Tubulin β was served as the loading control.

(I) TG and TC contents of primary mouse hepatocytes that were isolated from the WT female mice in the indicated group (n = 6).

In all statistical plots, data are expressed as the mean ± SD and analyzed by one-way ANOVA with Bonferroni analysis. The mRNA expression of target genes was normalized to that of Actb. ***p < 0.001; n.s., no significance, p ≥ 0.05.
